# Non-epigenetic induction of HEXIM1 by DNMT1 inhibitors and functional relevance

**DOI:** 10.1038/s41598-020-78058-y

**Published:** 2020-12-03

**Authors:** Vikas Sharma, Monica M. Montano

**Affiliations:** Department of Pharmacology, School of Medicine, Case Western Reserve University, 10900 Euclid Ave., Cleveland, OH 44106 USA

**Keywords:** Cancer, Oncology

## Abstract

We have been studying the role of Hexamethylene bisacetamide (HMBA) Induced Protein 1 (HEXIM1) as a tumor suppressor whose expression is decreased in breast and prostate cancer. The anti-cancer actions of HEXIM1 in melanomas and AML have been reported by other groups. Previous studies have shown that 5-Aza-2′deoxycytidine (5-AzadC), a DNMT1 inhibitor, induces re-expression of tumor suppressor genes by removing/erasing methylation marks from their promoters. Our studies highlighted another mechanism wherein 5-AzadC induced DNA damage, which then resulted in enhanced occupancy of NF-ĸB, P-TEFb, and serine 2 phosphorylated RNA Polymerase II on the *HEXIM1* gene. As a consequence, 5-AzadC induced HEXIM1 expression in prostate cancer cell lines and triple negative breast cancers. 5-AzadC-induced DNA damage enhanced P-TEFb occupancy via a mechanism that involved activation of ATR and ATM and induction of NF-ĸB recruitment to the *HEXIM1* promoter. Downregulation of NF-ĸB attenuated 5-AzadC-induced HEXIM1 expression in prostate and breast cancer cells. The functional relevance of 5-AzadC-induced HEXIM1 expression is revealed by studies showing the HEXIM1 is required for the induction of apoptosis. Collectively, our findings support a non-epigenetic mechanism for 5-AzadC-induced re-expression of HEXIM1 protein, and may contribute to the clinical efficacy of 5-AzadC.

## Introduction

Cancer development is strongly correlated with progressive accumulation of multiple genetic and epigenetic events, which may eventually lead to genetic instability and subsequently to the progressive disease. The most common epigenetic change in cancers is hypermethylation of the CpG islands within the promoters of tumor suppressor genes due to either increased activity or overexpression of DNA-methyltransferase 1 (DNMT1). Two nucleoside (cytosine) analogs 5-aza-2′-cytidine (5-AzaC) and 5-aza-2′-deoxycytidine (5-AzadC) have been characterized as DNMT inhibitors. These inhibitors have been approved by the US Food and Drug Administration (FDA) for the treatment of myelodysplastic syndrome (MDS) and acute myeloid leukemia (AML)^1^. The FDA approval of these “epidrugs” supports the therapeutic potential of DNMT1 inhibitors.

Despite the widely accepted demethylating activity of DNMT1 inhibitors^2–4^, another proposed basis for their cytotoxicity is the formation of irreversible, covalent enzyme–DNA adducts and the ensuing response to DNA damage^5,6^. The responses to 5-AzaC-induced DNA double-strand breaks (DSBs) are mediated predominantly via the Phosphatidylinositol 3-kinase-related kinase (PIKK) family member sensor proteins, Ataxia Telangiectasia, Mutated (ATM) and Ataxia Telangiectasia and Rad3-related protein (ATR)^7^. ATR and ATM then phosphorylate downstream Checkpoint Kinase 1 and 2 (CHK1 and CHK2) proteins, respectively, to further activate downstream targets. In particular, DNA-damage triggered ATM directly activates IKK and the NF-ĸB pathway. NF-ĸB has been reported to be involved in the recruitment of Positive Transcriptional Elongation Factor B (P-TEFb) to the promoter regions of genes involved in innate and adaptive immunity^8^. P-TEFb is a heterodimer between the cyclin-dependent kinase 9 (Cdk9) and its regulatory subunit Cyclin T1.

One of the promoter regions wherein P-TEFb is recruited to is the gene encoding the tumor suppressor Hexamethylene bisacetamide (HMBA) Induced Protein 1 (HEXIM1)^9^. HEXIM1, in turn, inhibits P-TEFb activity and thereby transcriptional elongation of HEXIM1 target genes^10^. Studies from our laboratory demonstrated that HEXIM1 is a co-repressor of the androgen and estrogen receptors, and is required for the inhibition of the activity of these receptors by anti-hormones^11,12^. HEXIM1 expression is decreased in hormone resistant breast and prostate cancer. HEXIM1 expression is also decreased in metastatic breast cancer, and re-expression of HEXIM1 resulted in the inhibition of mammary tumor growth and metastasis^13^. Together these studies support the re-expression of HEXIM1 as a therapeutic goal.

In this report, we show that 5-AzadC induced DNA damage and recruitment of CDK9 and serine 2 phosphorylated (S2P) RNAPII to the *HEXIM1* promoter and *HEXIM1* coding region, respectively. Occupancy of *HEXIM1* gene by P-TEFb results in increased HEXIM1 transcription. The resulting increase in HEXIM1 expression resulted in upregulation the expression of p21, likely mediated by HEXIM1 upregulation of p53 stability^14^. Thus, the induction of the tumor suppressor protein HEXIM1 is part of the cellular response to DNA damage and the resulting inhibition of cell cycle progression or apoptosis. Our findings also have important implications for the development of small molecules or other strategies to induce the expression of HEXIM1 as therapeutic options against cancer.

## Results

### 5-Aza-2′deoxycytidine induced HEXIM1 expression

**Figure 1:**
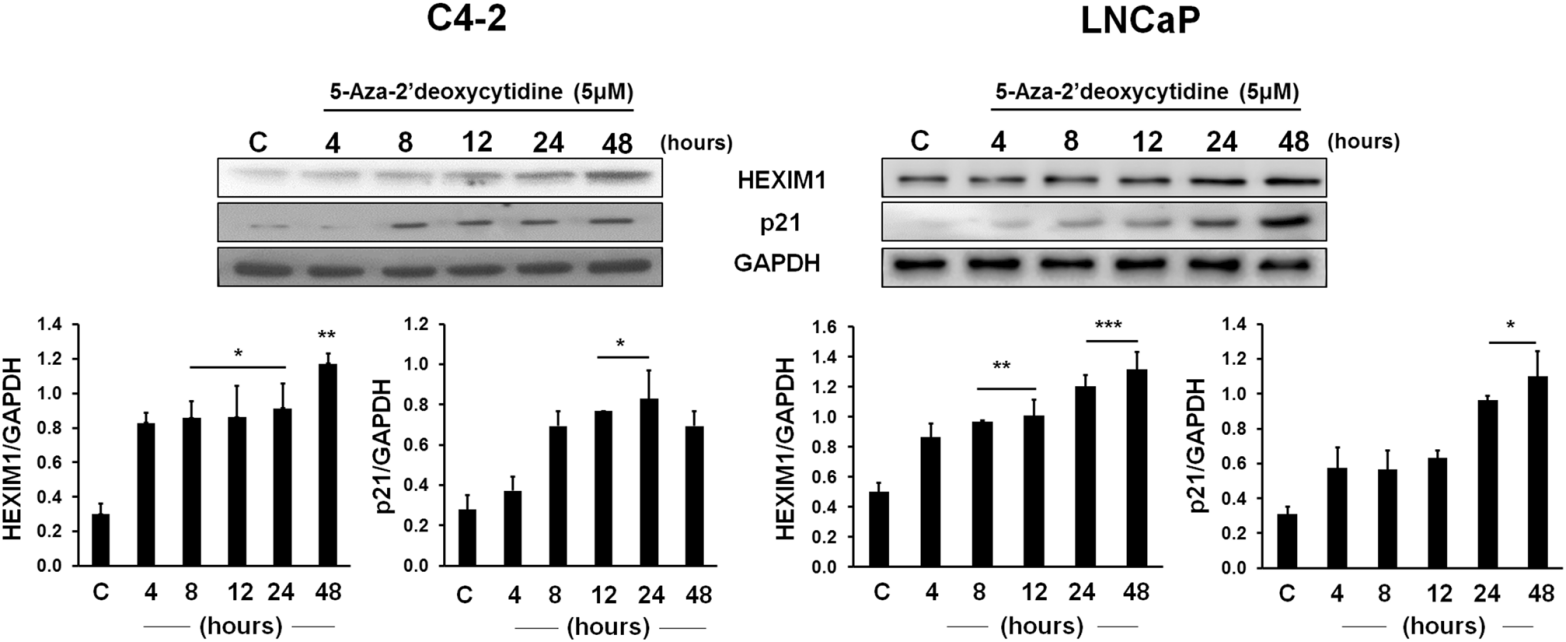
5-Aza-2′deoxycytidine induced HEXIM1 expression. C4-2 and LNCaP cells were treated with 5-AzadC (5 μΜ) at the indicated time points and the expression of HEXIM1 and p21 normalized to GAPDH expression were assessed using western blots. Represented are blots cut into strips prior to blotting to minimize the amounts of antibodies required. Figures are representative of at least 3 independent experiments. *P < 0.05, **P < 0.01, and ***P < 0.001 vs. Control.

Because of the well-known role of DNMT1 in the inhibition of the expression of tumor suppressor genes, we determined if DNMT1 inhibitors could be utilized to re-express HEXIM1 in cancer cells. To determine the optimal dose and duration for 5-AzadC-induced HEXIM1 re-expression, C4-2 and LNCaP cells were treated with 5-AzadC at different time points and doses (Fig. 1 and Supplemental Fig. 1B). The optimal dose of 5 µM for induction of HEXIM1 expression (Supplemental Fig. 1A) is similar to the dose others have reported as required for 5-AzadC inhibition of DNMT1 and the ensuing demethylation of promoter regions^15,16^. While 5-AzadC induced HEXIM1 mRNA and protein expression by 8 h, maximum induction was evident at 48 h (Fig. 1 and Supplemental Fig. 1A). The level of induction of HEXIM1 expression was higher in C4-2 due to lower basal HEXIM1 expression in these cell lines, as we have previously reported^11^. 5-AzadC treatment did not result in alterations in DNMT1 expression (Supplementary Fig. 1C). No significant increase in HEXIM expression was evident after treatment with other DNMT1 inhibitors, Fludarabine and Cladribine (Supplementary Fig. 1D). As a measure of the functional relevance of the induction of HEXIM1 expression by 5-AzadC, we examined the expression of p21, which was upregulated by HEXIM1 during HEXIM1-induced cancer cell differentiation^17^. 5-AzadC induced p21 expression by 8 h, and the maximum induction of p21 expression was observed 24–48 h after treatment in C4-2 and LNCaP cell lines (Fig. 1). Based on these results, the 48-h time point after 5-AzadC treatment was selected for subsequent experiments.

### Non-epigenetic mechanism for 5-Aza-2′deoxycytidine-induced HEXIM1 expression

**Figure 2:**
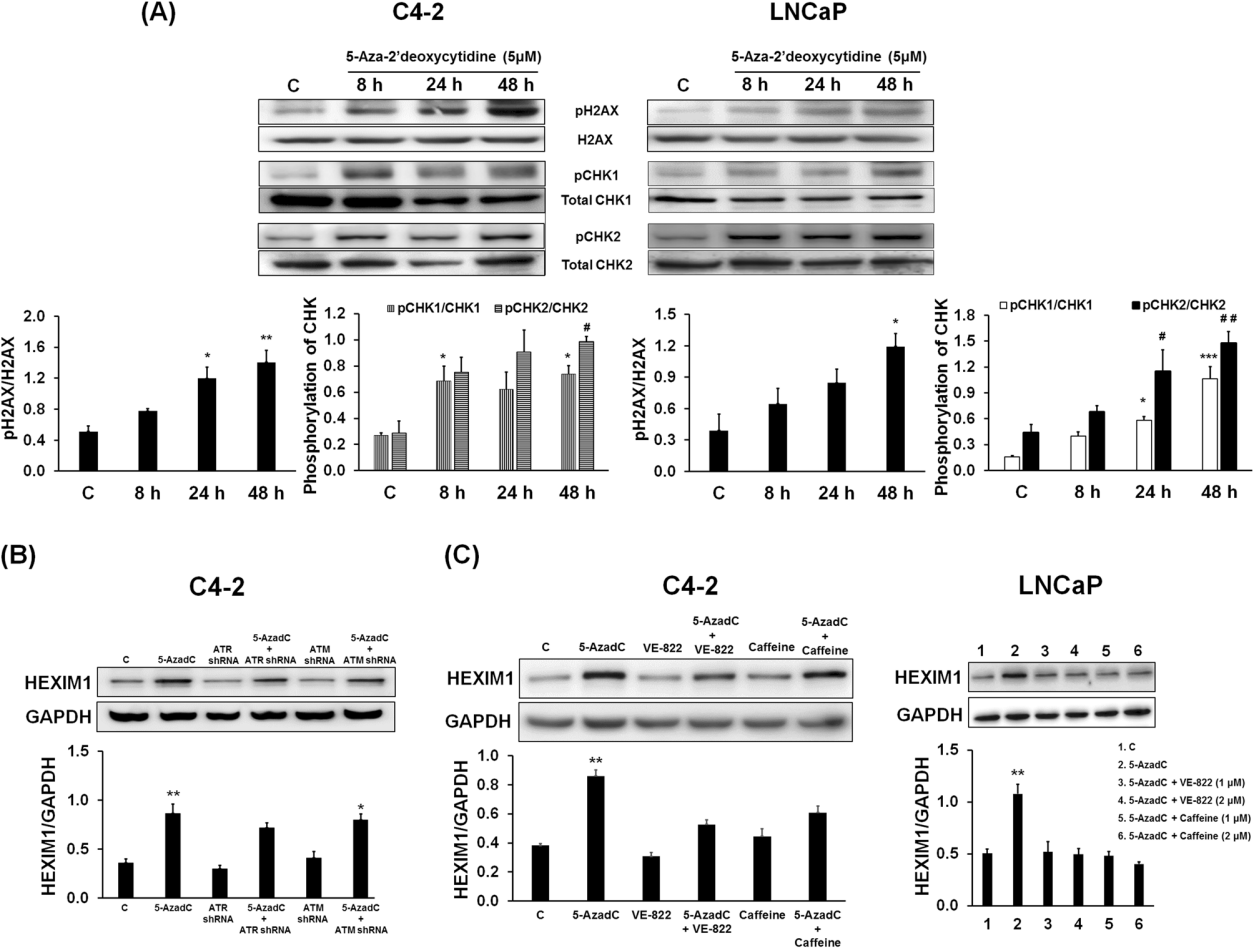
Involvement of activated ATR and ATM in the induction of HEXIM1 expression by 5-Aza-2′deoxycytidine. **(A)** C4-2 and LNCaP cells were treated with 5-AzadC (5 µM) at the indicated time points. Expression of phospho-CHK1, phospho-CHK2, and phospho-histone H2A.X normalized to total CHK1, total CHK2, and total histone H2A.X, respectively, were assessed by western blots. **(B)** C4-2 cells were infected with control, ATR, or ATM shRNA lentiviruses followed by puromycin selection. Cells were then treated with 5-AzadC (5 µM) for 48 h. Cell lysates were prepared and HEXIM1 and GAPDH proteins were analyzed by western blot. *P < 0.05 and **P < 0.01 vs. Control. Figures are representative of at least 3 independent experiments. **(C)** C4-2 and LNCaP cells were pre-treated with an ATR inhibitor, VE-822 (1 µM and 2 µM), or an ATM inhibitor, caffeine (1 µM and 2 µM) for 2 h, followed by 5-AzadC treatment (5 µM) for 48 h. Cell lysates were prepared and HEXIM1 and GAPDH proteins were analyzed by western blot. Represented are blots cut into strips prior to blotting to minimize the amounts of antibodies required. Figures are representative of at least 3 independent experiments. *P < 0.05, **P < 0.01, and ***P < 0.001 vs. Control. ^#^P < 0.05 and ^##^P < 0.01 vs. Control for pCHK2/CHK2.

While there is evidence for methylation of the *HEXIM1* promoter by DNMT1^18,19^, involvement of other mechanisms for DNMT1 inhibitor-induced HEXIM1 expression has not been reported. Previous studies have shown that 5-AzadC-induced DNA damage is associated with the phosphorylation of CHK1 and CHK2 by ATR and ATM kinases, respectively^7^. Involvement of a non-epigenetic mechanism should provide novel insight into the regulation of HEXIM1 expression in cancer cells. Consistent with 5-AzadC-induced SSB and/or DSB, 5-AzadC treatment resulted in increased levels of phosphorylated histone H2A.X, CHK1, and CHK2 proteins in C4-2 and LNCaP cells (Fig. 2A and Supplemental Fig. 1E). The time course for induction of pCHK1 and pCHK2 by 5-AzadC was similar to that observed for the induction of HEXIM1 expression. Involvement of the DNA damage response pathway in the upregulation of HEXIM1 expression by 5-AzadC was validated by downregulating the expression or activity of ATM or ATR. 5-AzadC-induced HEXIM1 expression was attenuated by downregulation of ATM by shRNAs (Fig. 2B and Supplemental Fig. 2A). Because of the modest attenuation of 5-AzadC-induced HEXIM1 expression by downregulating either ATR or ATM individually, we also utilized a pharmacological inhibitor of ATR (VE-822) and a pharmacological inhibitor of ATM (caffeine). While VE-822 and caffeine exhibit selectivity towards ATR and ATM, respectively, these compounds also exhibit some activity on the other PIKK family member sensor protein [ref.^20,21^ and Supplemental Fig. 2B]. 5-AzadC-induced HEXIM1 expression was attenuated by either VE-822 or caffeine (Fig. 2C). Decreases in the levels of pCHK1/CHK1 and pCHK2/CHK2 in the presence of either ATR and ATM inhibitors are shown in Supplemental Fig. 1B. These findings suggest that activation of the DNA damage response pathway by 5-AzadC is a contributing factor in the activation of downstream transcriptional factors that facilitates re-expression of the HEXIM1 protein.

### 5-Aza-2′deoxycytidine induced CDK9 recruitment and S2P RNAPII occupancy on the *HEXIM1* promoter

**Figure 3:**
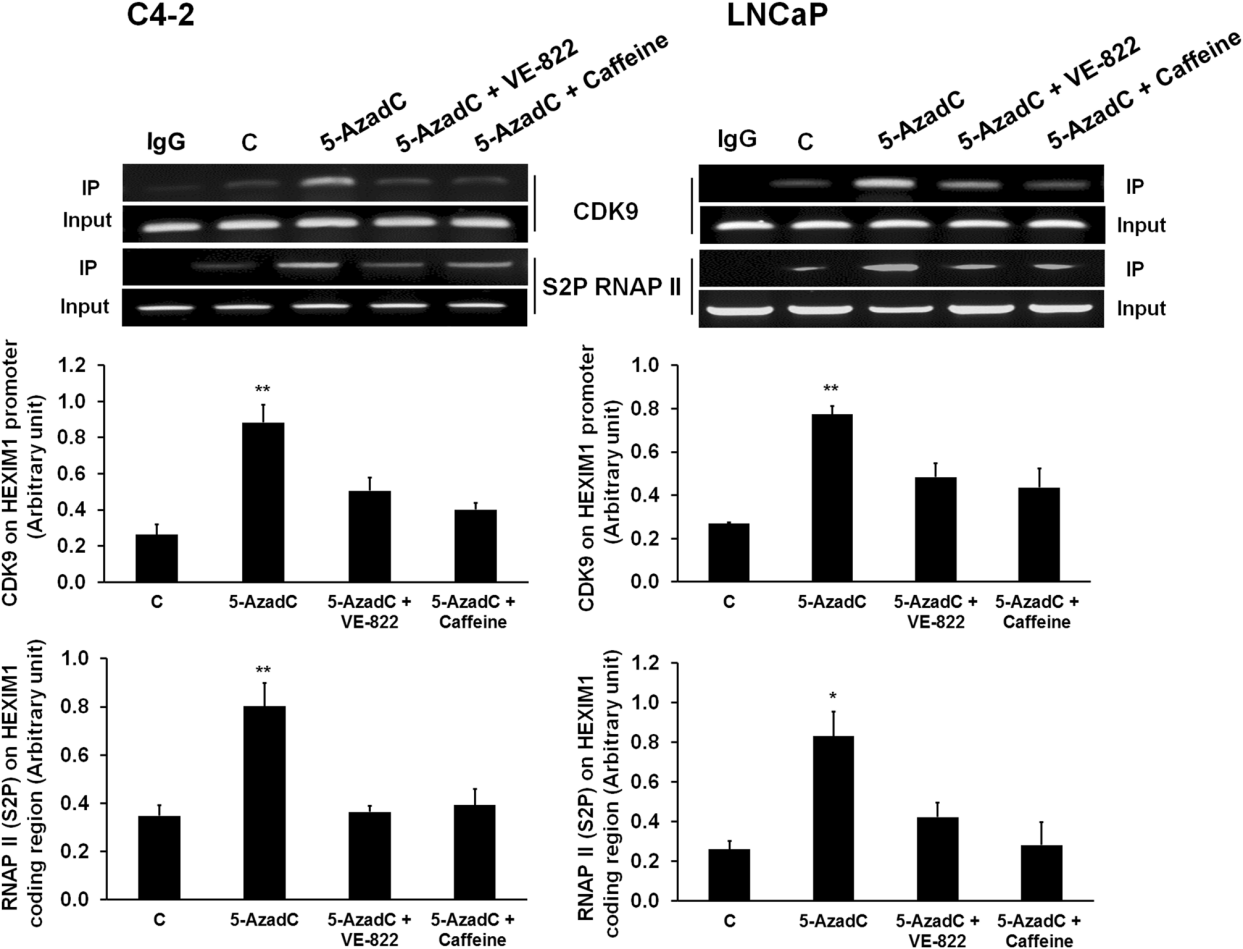
5-Aza-2′deoxycytidine induced CDK9 recruitment and S2P RNAPII occupancy on the *HEXIM1* promoter. C4-2 and LNCaP cells were treated with VE-822 (2 μM) or caffeine (2 μM) for 2 h, followed by 5-AzadC for 2 h. Cells were processed for ChIP analyses of the occupancy of CDK9 on the *HEXIM1* promoter and occupancy of serine 2 phosphorylated (S2P) RNAPII on the *HEXIM1* coding region. Input DNA was used as normalization control. IgG control was used as a negative control. Figures are representative of at least 3 independent experiments. *P < 0.05 and **P < 0.01 vs. Control.

We then determined how 5-AzadC-induced DNA damage results in upregulation of *HEXIM1* gene transcription. DNA damage has been reported to induce the release of P-TEFb from the promoter-bound 7SK snRNP complex^10,22^ and RNAPII pause release, resulting in transcriptional elongation and activation^8,23,24^. 5-AzadC has been reported to be a P-TEFb releasing agent^25^, but the mechanism was not clearly defined. Release of P-TEFb from the 7SK snRNP complex has been reported to result in activation of *HEXIM1* transcription^9^. Thus, we determined whether 5-AzadC-induced HEXIM1 re-expression can be attributed to enhanced recruitment of CDK9 to the *HEXIM1* promoter and the ensuing increase in S2P RNAPII occupancy on the *HEXIM1* coding region in C4-2 and LNCaP cells. As shown in Fig. 3, 5-AzadC induced CDK9 recruitment to the *HEXIM1* promoter, and this was attenuated in VE-822- or caffeine-treated cells. Similarly, 5-AzadC treatment resulted in enhanced occupancy of S2P RNAPII serine phosphorylation on the *HEXIM1* coding region, which can also be inhibited by VE-822 or caffeine (Fig. 3). CDK9 levels were not altered by treatment with 5-AzadC in the absence or presence of VE-822 or caffeine (Supplemental Fig. 3A). Collectively, our data suggest that 5-AzadC-induced DNA damage promotes binding of transcription factors associated with transcriptional elongation to the *HEXIM1* promoter, resulting in increased HEXIM1 expression.

### Nuclear factor-kappa B (NF-ĸB) is a mediator in 5-Aza-2′deoxycytidine-induced HEXIM1 expression

**Figure 4:**
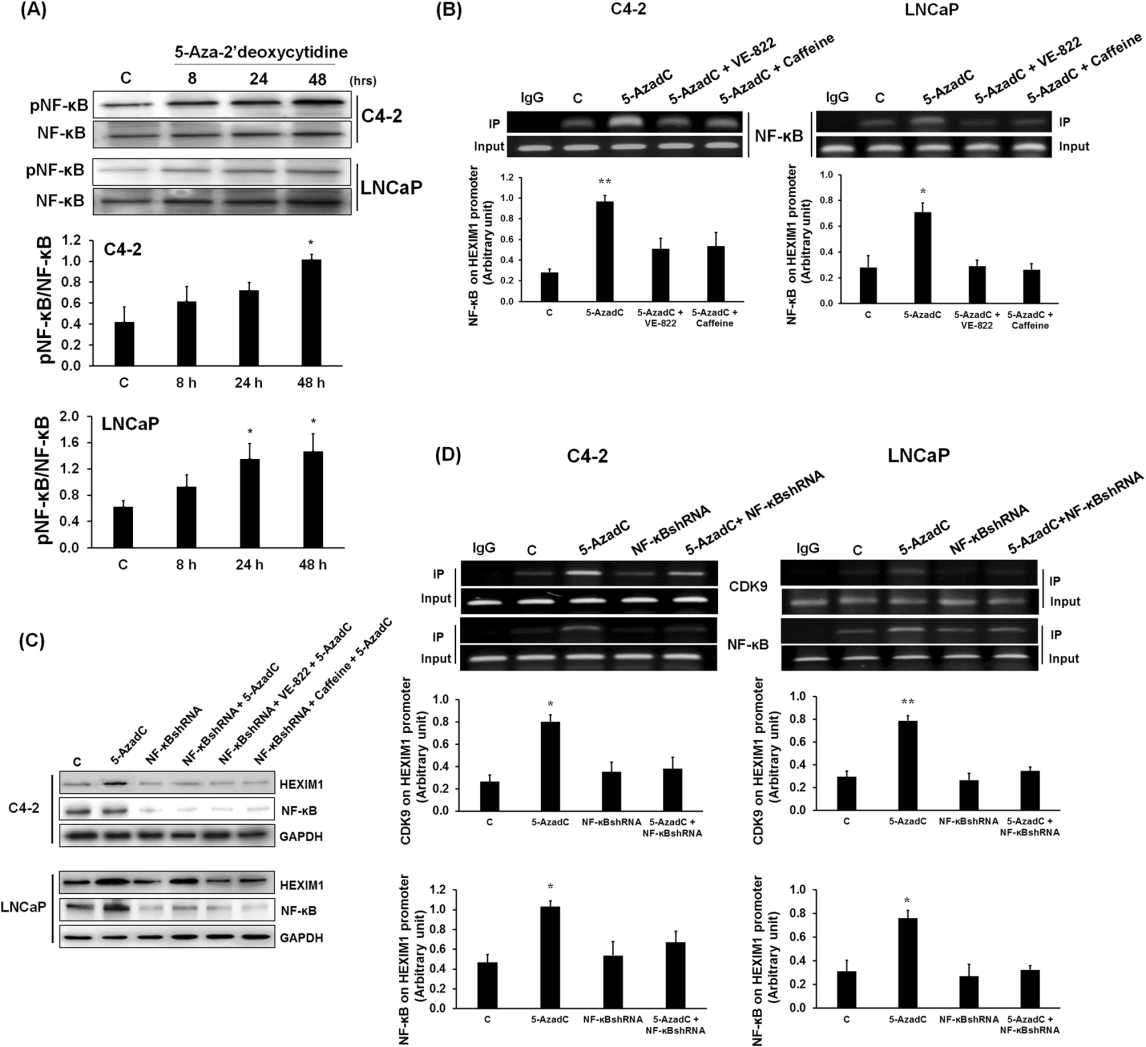
NF-ĸB is a mediator of 5-Aza-2′deoxycytidine-induced HEXIM1 expression. **(A)** LNCaP and C4-2 cells were treated with 5-AzadC. Cell lysates were prepared and expression of phospho-NF-ĸB and NF-ĸB were analyzed by western blots. Represented are blots cut into strips prior to blotting to minimize the amounts of antibodies required. Figures are representative of at least 3 independent experiments. *P < 0.05 vs. Control. **(B)** C4-2 and LNCaP cells were treated with 2 µM of VE-822 or caffeine for 2 h, followed by 5-AzadC (5 µM) for 2 h. Cells were processed for ChIP analyses of the occupancy of NF-ĸB on the *HEXIM1* promoter. Input DNA was used as normalization control. IgG control was used as a negative control. *P < 0.05 and **P < 0.01 vs. Control. **(C)** C4-2 and LNCaP cells were infected with lentiviruses expressing control or NF-ĸB shRNA, and selected with puromycin. Some cells were treated with 2 µM of VE-822 or caffeine for 2 h, followed by 5-AzadC (5 µM) for 2 h. Cell lysates were prepared and expression of indicated proteins were analyzed by western blot. Represented are blots cut into strips prior to blotting to minimize the amounts of antibodies required. Figures are representative of at least 3 independent experiments. **(D)** LNCaP and C4-2 cells were infected with control or NF-ĸB shRNA lentiviruses and selected with puromycin. Some cells were treated with 5-AzadC (5 μΜ, 2 h). Cells were then processed for ChIP analyses of recruitment of CDK9 and NF-ĸB to the *HEXIM1* promoter. Input DNA was used as normalization control. IgG control was used as a negative control. Figures are representative of at least 3 independent experiments. *P < 0.05 and **P < 0.01 vs. Control.

It has been reported that following DNA damage, NF-ĸB is directly or indirectly involved in the dissociation of P-TEFb from an inhibitory complex with 7SK snRNP, resulting in transcriptional activation of certain genes^10,26^. NF-ĸB is phosphorylated at Ser 276 in response to DSB, a process that requires ATM^8^. Consistent with 5-AzadC-induced DSB and activation of ATM, we observed upregulation of phospho-Ser 276 NF-ĸB in response to 5-AzadC treatment (Fig. 4A).

We then examined the relative role of NF-ĸB in 5-AzadC-induced recruitment of CDK9 to the *HEXIM1* promoter. In C4-2 and LNCaP cells, 5-AzadC induced NF-ĸB recruitment to the *HEXIM1* promoter, which is attenuated by VE-822 or caffeine (Fig. 4B). NF-ĸB recruitment to the *HEXIM1* coding region was not altered by 5-AzadC (Supplemental Fig. 3B). NF-ĸB levels were not altered by treatment with 5-AzadC in the absence or presence of VE-822 or caffeine (Supplemental Fig. 3C).

The critical role of NF-ĸB in 5-AzadC-induced HEXIM1 expression is supported by attenuation of 5-AzadC-induced HEXIM1 expression upon knockdown of NF-ĸB in LNCaP and C4-2 cells (Fig. 4C and Supplementary Fig. 4A). Vehicle-treated (control) cells expressing NF-ĸB shRNA did not exhibit altered HEXIM1 expression. However, knockdown of NF-ĸB in LNCaP cells did not result in significant attenuation of 5-AzadC-induced HEXIM1 expression (Fig. 4C), suggesting that there are factors expressed in LNCaP cells that can compensate for downregulation of NF-ĸB. We further defined the role of NF-ĸB in the transcriptional regulation of the *HEXIM1* gene. Knockdown of NF-ĸB and the ensuing decrease in promoter occupancy of NF-ĸB resulted in attenuation of 5-AzadC-induced recruitment of CDK9 to the *HEXIM1* promoter (Fig. 4D). CDK9 levels were not altered upon knockdown of NF-ĸB (Supplemental Fig. 4B).

### The relative role of HEXIM1 in the induction of apoptosis and inhibition of cell proliferation by 5-Aza-2′deoxycytidine

**Figure 5:**
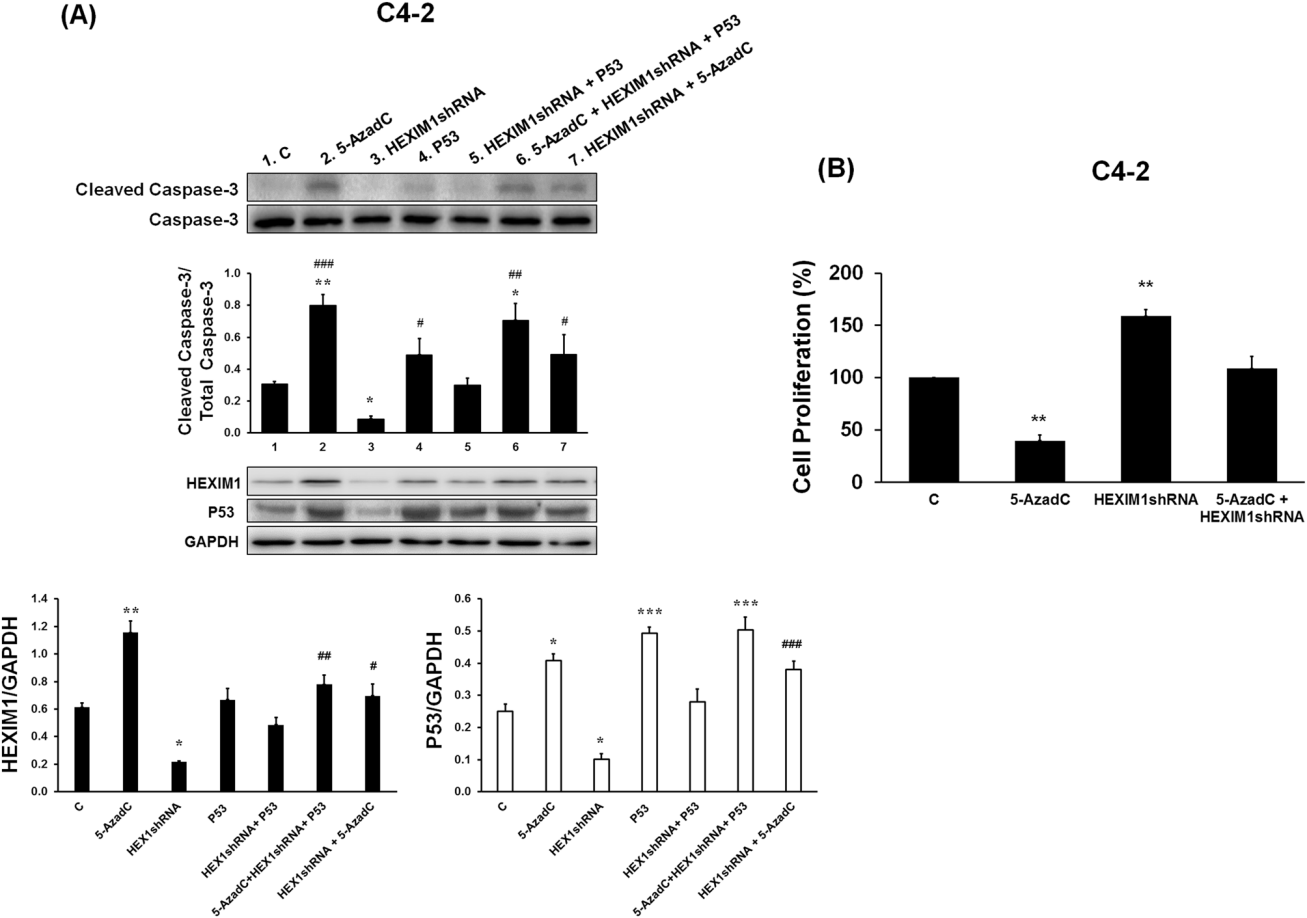
HEXIM1 is required for 5-Aza-2′deoxycytidine-induced apoptosis and inhibition of cell proliferation by 5-Aza-2′deoxycytidine. **(A)** C4-2 cells were infected with control or HEXIM1shRNA lentiviruses followed by puromycin selection. Some cells were transfected with p53 expression vector and/or treated with 5-AzadC (5 μΜ, 48 h). Cell lysates were prepared and expression of indicated proteins were analyzed by western blot. Represented are blots cut into strips prior to blotting to minimize the amounts of antibodies required. Figures are representative of at least 3 independent experiments. *P < 0.05 and **P < 0.01, ***P < 0.001 versus Control, and ^#^P < 0.05, ^##^P < 0.01, and ^###^P < 0.001 vs. HEXIM1shRNA. **(B)** C4-2 cells were infected with control or HEXIM1shRNA-containing lentiviruses followed by selection using puromycin and plating for cell proliferation. Some cells were treated with 5-AzadC. After 5 days, WST-1 solution (10 mg/ml) was added to each well. The formazan crystals formed inside the viable cells were measured at 450 nm using a microplate reader. Figures are representative of at least 3 independent experiments **P < 0.01 vs. Control.

We then examined the functional relevance of 5-AzadC-induced HEXIM1 expression. In particular, we determined the relative role of HEXIM1 in 5-AzadC-induced apoptosis, and if this role involved the reported HEXIM1-induced increases in p53 levels^27^. Treatment with 5-AzadC resulted in increased levels of cleaved caspase-3 in C4-2 cells (Fig. 5A). Downregulation of HEXIM1 resulted in attenuated 5-AzadC-induced increase in cleaved caspase-3 levels, although this could be attributed to the decrease in basal levels of cleaved caspase-3 observed in vehicle-treated/HEXIM1shRNA-transfected cells (Fig. 5A). Transfection of expression vector for p53 into HEXIM1 shRNA-transfected cells partially restored cleaved caspase 3 levels to that observed in control transfected 5-AzadC-treated cells. Downregulation of HEXIM1 also attenuated 5-AzadC-induced decrease in proliferation of C4-2 cells (Fig. 5B). We examined if 5-AzadC induced other types of cell death, but we did not observe an increase in the levels of a marker for necroptosis, phosphorylated MLKL (Supplementary Fig. 5).

### 5-Aza-2′deoxycytidine-induced HEXIM1 expression in triple negative breast cancer (TNBC) is also mediated by NF-ĸB and HEXIM1 is critical for 5-Aza-2′deoxycytidine-induced apoptosis

We have also been studying the HEXIM1 as a breast tumor suppressor whose expression is decreased in tamoxifen resistant, triple negative, and metastatic breast cancer^13,28–30^. HEXIM1 inhibits metastasis by inhibiting cell invasion, angiogenesis, and the premetastatic niche^13,28^. Thus, HEXIM1 induction is also a potential therapeutic approach for breast cancer.

**Figure 6:**
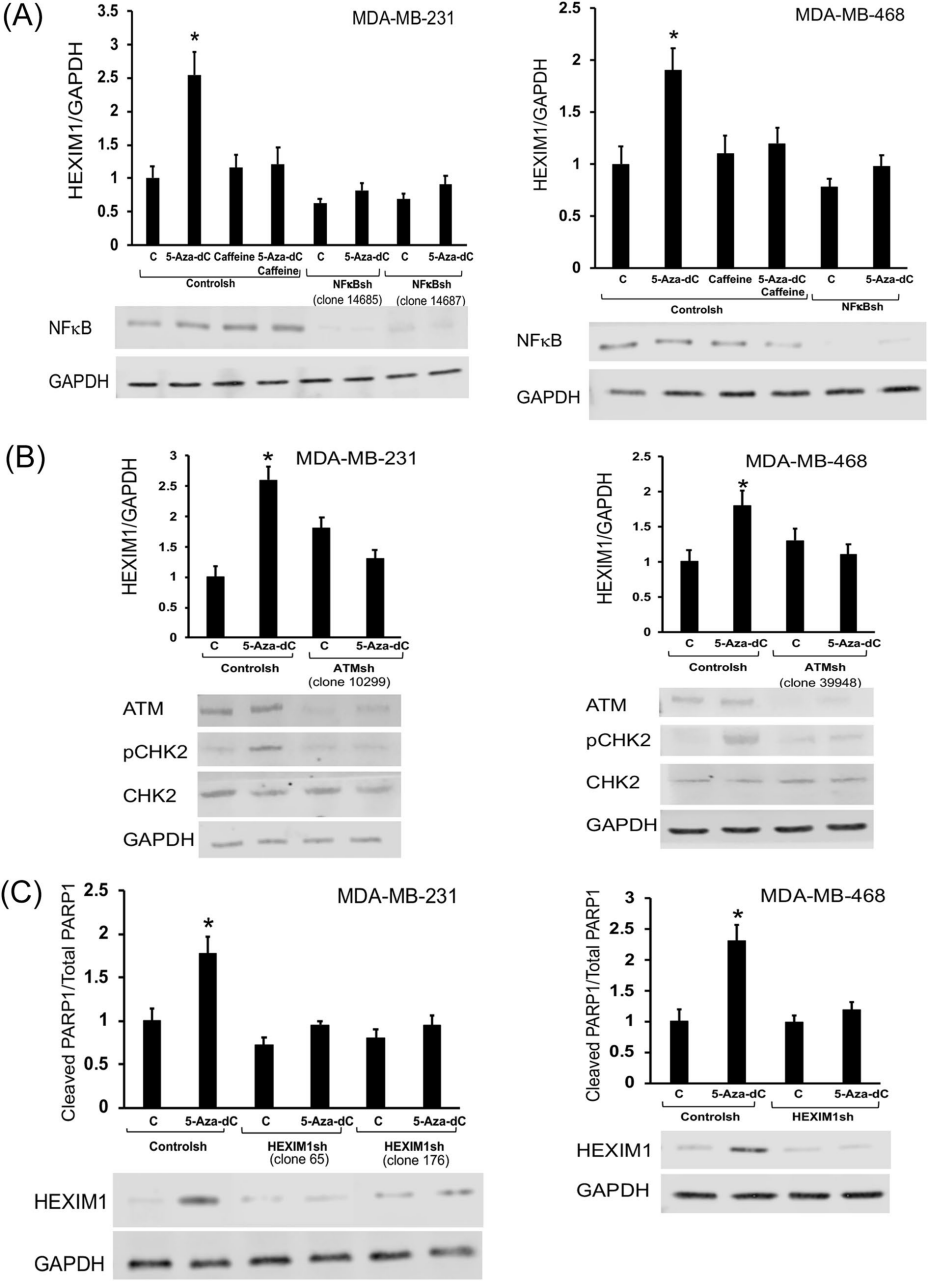
5-Aza-2′deoxycytidine-induced HEXIM1 expression in triple negative breast cancer is mediated by ATM and NF-ĸB, and HEXIM1 is critical for 5-Aza-2′deoxycytidine-induced apoptosis. **(A)** MDA-MB-231 and MDA-MB-468 cells were infected with control or NF-ĸB shRNA lentiviruses followed by puromycin selection or pre-treated with ATM inhibitor, caffeine (2 µM). Some cells were then treated with 5-AzadC (5 µM) for 48 h. **(B)** MDA-MB-231 and MDA-MB-468 cells were infected with control or ATM shRNA lentiviruses followed by puromycin selection. Some cells were then treated with 5-AzadC (5 µM) for 48 h. **(C)** MDA-MB-231 were infected with control or HEXIM1shRNA lentiviruses followed by puromycin selection. Some cells were treated with 5-AzadC (5 μΜ, 48 h). For (**A**),(**B**), and (**C**) cell lysates were prepared and expression of indicated proteins were analyzed by western blot. Represented are blots cut into strips prior to blotting to minimize the amounts of antibodies required. *P < 0.01 versus Control. Figures are representative of at least 3 independent experiments.

5-AzadC induced HEXIM1 expression in three TNBC lines, MDA-MB-231, MDA-MB-468, and MDA-MB-453 (Fig. 6A and Supplemental Fig. 6A). We also observed 5-AzadC-induced HEXIM1 expression in breast cancer cells representative of the luminal subtype, MCF7 and T47D (Supplemental Fig. 6B). A similar mechanism as observed in prostate cancer cells involving induction of DNA damage is supported by the attenuation of the induction of HEXIM1 expression in TNBC cells in the presence of caffeine or NF-ĸB shRNA (Fig. 6 and Supplemental Fig. 6A). Further support for the aforementioned mechanism is the attenuation of 5-AzadC-induced HEXIM1 expression after downregulating ATM expression (Fig. 6B). The increase in pCHK2 levels in 5-AzadC treated cells was attenuated by transfecting cells with ATM shRNA or by caffeine treatment (Fig. 6B and Supplementary Fig. 6C). Our results are also consistent with an important role for HEXIM1 in 5-AzadC-induced apoptosis (as assessed by levels of cleaved PARP1/PARP1, Fig. 6C). However, the expression of mutant p53 in MDA-MB-231 and MDA-MB-468 suggests this action of HEXIM1 is wild type p53-independent.

## Discussion

The biological actions of DNMT1 inhibitors have been attributed to two mechanisms. The first mechanism is reversal of epigenetic aberrations induced by DNMT^31,32^. The second mechanism is the induction of DNA damage and the formation of irreversible, covalent enzyme–DNA adducts. We determined that 5-AzadC-induced DNA damage resulted in upregulation of the tumor suppressor HEXIM1, and established a role for the DNA damage response pathway in the transcriptional regulation of *HEXIM1* in prostate and breast cancer cells. 5-AzadC treatment induced the recruitment of CDK9, a transcription elongation factor, to the *HEXIM1* promoter. As a consequence, we observed increased occupancy of S2P RNAPII on the *HEXIM1* coding region, a marker for the release of RNAPII pausing. The attenuation of CDK9 recruitment upon inhibition of ATR and ATM, is consistent with the involvement of checkpoint kinases in 5-AzadC-induced HEXIM1 expression.

Our laboratory has invested considerable effort in the identification of agents that can induce HEXIM1 expression. In addition to unique HEXIM1 inducers like the compound 4a1^17^ we have determined that inhibitors of the histone demethylase, KDM5B, also upregulated HEXIM1 expression^33^. 5-AzadC, as well as other agents that induce release P-TEFb, have been reported to upregulate *HEXIM1* gene transcription^9,25^. While 5-AzadC has also been reported to be a P-TEFb releasing agent that can activate a *HEXIM1* promoter luciferase reporter^25^, the mechanism and consequences were not clearly defined.

DNA damage has been reported to induce the release of P-TEFb from the promoter-bound 7SK snRNP complex^10,22^ and RNAPII pause release, resulting in transcriptional elongation and activation^8,23,24^. NF-ĸB is a key molecular player that promotes the recruitment of CDK9 and the transcriptional activation of the *HEXIM1* gene. The relative role of NF-ĸB in the activation of *HEXIM1* gene transcription was assessed via shRNA-mediated knockdown of NF-ĸB, which resulted in attenuated 5-AzadC-induced recruitment of CDK9 to the *HEXIM1* gene. NF-ĸB plays critical roles in cellular stress response and modulates transactivation of a large number of genes that participate in various cellular processes involved in DDR^34^. Along this line, treatment with Tumor necrosis factor (TNF) resulted in the activation of ATM, which was then involved in the proteasomal degradation of IKB and the ensuing release of NF-ĸB^8^. NF-ĸB then promoted the recruitment of PPMiG/PP2Cy phosphatase to their target promoters, resulting in the release of P-TEFb from the inhibitory 7SK snRNP complex and phosphorylation of serine 2 of RNAPII^10,26^. In our study, 5-AzadC-induced DNA damage promoted recruitment of NF-ĸB, along with the CDK9 and S2P RNAPII occupancy, on the *HEXIM1* promoter to induce *HEXIM1* gene transcription. Downregulation of ATM/ATR expression or inhibition of their kinase activity resulted in the attenuation of NF-ĸB recruitment and/or *HEXIM1* gene transcription and expression. NF-ĸB-mediated induction of the expression of HEXIM1 underscores the divergent role of NF-ĸB in cancer, with reports supporting not only its canonical role as a tumor promoter but also its role as a tumor suppressor^35,36^.

Our studies also provide further insight into consequences of 5-AzadC-induced DNA damage by showing that HEXIM1 plays a critical role in 5-AzadC-induced apoptosis and cell proliferation. HEXIM1 upregulates the stability of another tumor suppressor, p53 via an interaction between HEXIM1 and p53^14^. The implication from the present study is that HEXIM1 is another mediator in the upregulation of p53 and p21 resulting from DNA damage and the induction of cell cycle arrest or apoptosis. However, the role of HEXIM1 in 5-AzadC-induced apoptosis appears to be wild type p53-independent in TNBC. Both MDA-MB-231 and MDA-MB-468 express mutant p53. This is critical because p53 is the second most common alteration in CRPC^37,38^ and 60–80% of TNBC expressing mutated p53^39,40^. Wild type p53-independent induction of apoptosis, coupled with downregulation of factors with well-established roles in tumor progression and metastasis by HEXIM1^17,41^, makes re-expression of HEXIM1 via DNMT1 inhibitors an attractive anticancer strategy.

## Materials and methods

### Cell lines, cell culture and treatment

Prostate cancer cell lines and triple negative breast cancer cell lines were obtained from American Tissue Culture Collection (ATCC) in April 2017. Cells were utilized within 8 passages of the stock obtained from ATCC, and were cultured in RPMI or DMEM media supplemented with 10% fetal bovine serum and antibiotics. Cells were maintained at 37 °C in an incubator (Thermo Fisher Scientific) containing 5% CO_2_ and 95% humidity. Cells seeded in plates were treated with 5-Aza-2′deoxycytidine (DNMT1 inhibitor), VE-822 (ATR inhibitor), and Caffeine (ATM inhibitor) at different time points and concentrations and then subjected to further experimentation.

### Western blotting analyses

Total protein was extracted from cells and subjected to western blot analyses as previously described^33^. Blots were cut into strips to minimize the amounts of antibodies required. Western blots were incubated using primary antibodies from Cell Signaling Technology [anti-ATR (cat. no. 13934), anti-ATM (cat. no. 2873), anti-phospho-CHK1 (cat. no. 2348), anti-phospho-CHK2 (cat. no. 2661), and anti-CHK2 (cat. no. 2662), anti-phospho-histone H2A.X Ser 139 (cat. no. 9718), anti-histone H2A.X (cat. no. 7631), anti-phospho-MLKL (cat. no. 91689), anti-MLKL (cat. no. 14993), and anti-p53 (cat. no. 9282)], from Santa Cruz Biotechnology [anti-Caspase 3 (cat. no. sc-271028), anti-CHK1 (cat. no. sc-56291), anti-DNMT1 (cat. no. sc-271729), anti-NF-ĸB (cat. no. sc-166588), anti-p21 (cat. no. sc-397)], from Invitrogen [anti-phospho-NF-ĸB Ser 276 (cat. no. PA5-37718)], and from Sigma-Aldrich [anti-GAPDH (cat. no. MAB374)]. Anti-HEXIM1 is an in-house antibody^42^. Blots were then incubated with anti-rabbit or anti-mouse secondary antibody (cat. nos. 31460 and 31430, respectively, Thermo Scientific) at room temperature. The immunoblots were detected using SuperSignal™ West Femto Maximum Sensitivity Substrate (Thermo Scientific) and then visualized using the LI-COR Odyssey System (LI-COR Biosciences, Lincoln, NE, USA), and quantified using the Image J software.

### RT-PCR

C4-2 cells were treated with 5-AzadC at the indicated concentration for 8, 24, and 48 h. Total RNA was extracted using Trizol- RNA isolation protocol according to the manufacturer’s instructions (Invitrogen) and 2 µg of RNA was reverse-transcribed into complementary DNA, which was amplified using Reverse- Transcriptase (RT)—PCR. The primer sequences used to measure the expression of HEXIM1 and GAPDH are given below. The mRNA expression levels of HEXIM1 gene were normalized to the expression level of the housekeeping gene GAPDH.

hHEX1 fw: ATGGCCGAGCCATTCTTGTCAG

hHEX1 rv: GTACGGTTTCCAATGCCGCTT

hGAPDH fw: GTCATCATCTCTGCCCCCTCTGCT

hGAPDH rv: CTTCTTGATGTCATCATATTTG.

### Chromatin Immunoprecipitation (ChIP)

ChIP assays were performed to assess recruitment of CDK9 and NF-ĸB to the promoter region or coding region of *HEXIM1* and recruitment of Serine 2 Phosphorylated RNAPII to the *HEXIM1* coding region. C4-2 and LNCaP cells were grown in 15 cm^2^ plates. C4-2 and LNCaP cells were transduced with control or NF-ĸB shRNA and/or treated with 5-AzadC in the absence or presence of VE-822 or Caffeine. Thereafter, cells were processed for ChIP analyses as described previously^42^. ChIP experiments were done using following antibodies: Normal mouse IgG (cat. no. sc-2025; Santa Cruz Biotechnology), anti-mouse IgM (cat. no. M8644; Sigma-Aldrich), anti-CDK9 (cat no. sc-13130; Santa Cruz Biotechnology), anti-RNAPII S2P (clone H5, cat. no. MMS-129R; Covance Research Products), and anti-NF-ĸB (cat. no. sc-166588; Santa Cruz Biotechnology). The following primers were used to amplify the *HEXIM1* gene regions:

*HEXIM1* promoter:

- 5′-GGGCCCGAAAGATAAGAACT-3′
- 5′-CTTCCCACAGCTCCTCTTCC-3′

*HEXIM1* coding region:

- 5′-ATGGCCGAGCCATTCTTGTCAG-3′
- 5′-GTACGGTTTCCAATGCCGCTT-3′

### Cell transduction and transfection

Lentiviral-mediated delivery of shRNAs was used to knockdown NF-ĸB, HEXIM1, ATM, or ATR expression in cells. Lentiviral particles were generated by transfecting HEK293FT cells with NF-ĸB shRNA, HEXIM1 shRNA, ATM shRNA, or ATR shRNA expression plasmids (Sigma-Aldrich) along with two other plasmids, envelope expressing plasmid (pMD2.G), and packaging plasmid (psPAX2), using Lipofectamine 3000. Forty to sixty hours after of transfection, medium containing lentivirus was collected. C4-2 and LNCaP cells were transduced with lentiviral particles in the presence of polybrene for 18–24 h. Transduced cells were selected using puromycin, harvested, and processed for immunoblotting to confirm knockdown of targeted proteins.

Some cells were transfected with expression vector for p53 (CMV5-p53) using Lipofectamine 3000.

### Proliferation assay

Cells were plated onto a 96-well plate at a density of 3000 cells/well. Some cells were infected with control shRNA or HEXIM1 shRNA lentiviruses for 48 h prior to plating onto 96-well plates for proliferation assays. Media containing DMSO or 5-Aza-2′deoxycytidine was replaced every other day. After 6 days, cell proliferation was assessed using the WST-1 assay. Absorbance was measured at 450 nm using a Molecular Devices plate reader (San Jose, CA).

### Statistical analyses

Statistical significance was determined using Student’s *t* test comparison. For some comparisons, probability values for the observed differences between groups were based on one-way ANOVA^43^. A probability (*p*) value of < 0.05 was accepted as an appropriate level of significance.

## Supplementary information

Supplementary Figures.

## Data Availability

The authors declare that all the materials, data and associated protocols related to this manuscript are available upon reasonable request.

## References

[1] Mack GS (2006). Epigenetic cancer therapy makes headway. J. Natl. Cancer Inst..

[2] Bender CM, Zingg JM, Jones PA (1998). DNA methylation as a target for drug design. Pharm. Res..

[3] Jones PA, Taylor SM, Wilson VL (1983). Inhibition of DNA methylation by 5-azacytidine. Recent Results Cancer Res..

[4] Taylor SM, Constantinides PA, Jones PA (1984). 5-Azacytidine, DNA methylation, and differentiation. Curr. Top. Microbiol. Immunol..

[5] Ferguson AT (1997). Role of estrogen receptor gene demethylation and DNA methyltransferase.DNA adduct formation in 5-aza-2'deoxycytidine-induced cytotoxicity in human breast cancer cells. J. Biol. Chem..

[6] Juttermann R, Li E, Jaenisch R (1994). Toxicity of 5-aza-2'-deoxycytidine to mammalian cells is mediated primarily by covalent trapping of DNA methyltransferase rather than DNA demethylation. Proc. Natl. Acad. Sci. USA.

[7] Kiziltepe T (2007). 5-Azacytidine, a DNA methyltransferase inhibitor, induces ATR-mediated DNA double-strand break responses, apoptosis, and synergistic cytotoxicity with doxorubicin and bortezomib against multiple myeloma cells. Mol. Cancer Ther..

[8] Fang L (2014). ATM regulates NF-kappaB-dependent immediate-early genes via RelA Ser 276 phosphorylation coupled to CDK9 promoter recruitment. Nucl. Acids Res..

[9] Liu P (2014). Release of positive transcription elongation factor b (P-TEFb) from 7SK small nuclear ribonucleoprotein (snRNP) activates hexamethylene bisacetamide-inducible protein (HEXIM1) transcription. J. Biol. Chem..

[10] McNamara RP, McCann JL, Gudipaty SA, D'Orso I (2013). Transcription factors mediate the enzymatic disassembly of promoter-bound 7SK snRNP to locally recruit P-TEFb for transcription elongation. Cell Rep..

[11] Yeh IJ (2014). HEXIM1 plays a critical role in the inhibition of the androgen receptor by anti-androgens. Biochem. J..

[12] Ketchart W (2011). HEXIM1 is a critical determinant of the response to tamoxifen. Oncogene.

[13] Ketchart W (2013). Inhibition of metastasis by HEXIM1 through effects on cell invasion and angiogenesis. Oncogene.

[14] Lew QJ (2012). Identification of HEXIM1 as a positive regulator of p53. J. Biol. Chem..

[15] Sharma V (2016). Sensitization of androgen refractory prostate cancer cells to anti-androgens through re-expression of epigenetically repressed androgen receptor - Synergistic action of quercetin and curcumin. Mol. Cell. Endocrinol..

[16] Sharma V (2016). Disulfiram and its novel derivative sensitize prostate cancer cells to the growth regulatory mechanisms of the cell by re-expressing the epigenetically repressed tumor suppressor-estrogen receptor beta. Mol. Carcinog..

[17] Ketchart W (2016). Induction of HEXIM1 activities by HMBA derivative 4a1: Functional consequences and mechanism. Cancer Lett..

[18] Sun D, Yu Q, Li P, Shen J (2016). Genomewide analyses of pathogenic and regulatory T cells of NOD mice reveal a significant difference in DNA methylation on chromosome X. J. Genet..

[19] Tan JL (2016). Stress from nucleotide depletion activates the transcriptional regulator HEXIM1 to suppress melanoma. Mol. Cell.

[20] Fokas E (2012). Targeting ATR in vivo using the novel inhibitor VE-822 results in selective sensitization of pancreatic tumors to radiation. Cell Death Disease.

[21] Sarkaria JN (1999). Inhibition of ATM and ATR kinase activities by the radiosensitizing agent, caffeine. Can. Res..

[22] Aj CQ, Bugai A, Barboric M (2016). Cracking the control of RNA polymerase II elongation by 7SK snRNP and P-TEFb. Nucl. Acids Res..

[23] Barboric M, Nissen RM, Kanazawa S, Jabrane-Ferrat N, Peterlin BM (2001). NF-kappaB binds P-TEFb to stimulate transcriptional elongation by RNA polymerase II. Mol. Cell.

[24] Bunch H (2015). Transcriptional elongation requires DNA break-induced signalling. Nat. Commun..

[25] Fujinaga K, Luo Z, Schaufele F, Peterlin BM (2015). Visualization of positive transcription elongation factor b (P-TEFb) activation in living cells. J. Biol. Chem..

[26] Gudipaty SA, McNamara RP, Morton EL, D'Orso I (2015). PPM1G binds 7SK RNA and Hexim1 to block P-TEFb assembly into the 7SK snRNP and sustain transcription elongation. Mol. Cell Biol..

[27] Lew QJ, Chu KL, Chia YL, Cheong N, Chao SH (2013). HEXIM1, a new player in the p53 pathway. Cancers (Basel).

[28] Ogba N (2010). HEXIM1 modulates vascular endothelial growth factor expression and function in breast epithelial cells and mammary gland. Oncogene.

[29] Wittmann BM, Fujinaga K, Deng H, Ogba N, Montano MM (2005). The breast cell growth inhibitor, estrogen down regulated gene 1, modulates a novel functional interaction between estrogen receptor alpha and transcriptional elongation factor cyclin T1. Oncogene.

[30] Wittmann BM, Wang N, Montano MM (2003). Identification of a novel inhibitor of cell growth that is down-regulated by estrogens and decreased in breast tumors. Cancer Res..

[31] Jarrard DF (1998). Methylation of the androgen receptor promoter CpG island is associated with loss of androgen receptor expression in prostate cancer cells. Cancer Res..

[32] Nakayama T (2000). Epigenetic regulation of androgen receptor gene expression in human prostate cancers. Lab. Invest..

[33] Montano MM (2019). Inhibition of the histone demethylase, KDM5B, directly induces re-expression of tumor suppressor protein HEXIM1 in cancer cells. Breast Cancer Res.: BCR.

[34] Wang W, Mani AM, Wu ZH (2017). DNA damage-induced nuclear factor-kappa B activation and its roles in cancer progression. J. Cancer Metastasis Treat..

[35] Perkins ND (2004). NF-kappaB: tumor promoter or suppressor?. Trends Cell Biol..

[36] Xia L (2018). Role of the NFkappaB-signaling pathway in cancer. Oncol. Targets Therapy.

[37] Quigley DA (2018). Genomic hallmarks and structural variation in metastatic prostate cancer. Cell.

[38] Robinson D (2015). Integrative clinical genomics of advanced prostate cancer. Cell.

[39] Shah SP (2012). The clonal and mutational evolution spectrum of primary triple-negative breast cancers. Nature.

[40] Podo F (2010). Triple-negative breast cancer: present challenges and new perspectives. Mol. Oncol..

[41] Chen R, Yik JH, Lew QJ, Chao SH (2014). Brd4 and HEXIM1: multiple roles in P-TEFb regulation and cancer. Biomed. Res. Int..

[42] Wittmann BM, Wang N, Montano MM (2003). Identification of a novel inhibitor of breast cell growth that is down-regulated by estrogens and decreased in breast tumors. Cancer Res..

[43] Yeh IJ, Ogba N, Bensigner H, Welford SM, Montano MM (2013). HEXIM1 down-regulates hypoxia-inducible factor-1alpha protein stability. Biochem. J..

