# Supplementary material for: Non-epigenetic induction of HEXIM1 by DNMT1 inhibitors and functional relevance
Source: Sci Rep. 2020 Dec 3;10:21015. doi: 10.1038/s41598-020-78058-y (PMC7713402; doi:10.1038/s41598-020-78058-y)
Supplement: Supplementary file 1 — Supplementary Figures. [file 41598_2020_78058_MOESM1_ESM.pdf]

Non-epigenetic induction of HEXIM1 by DNMT1 inhibitors and functional relevance

Vikas Sharma[a], Monica M. Montano[a]

Supplemental Figure 1. 5-AzadC dose dependent increase in the expression of HEXIM1, pCHK1, and pCHK2. C4-2 cells were treated with **(A)** 5-AzadC (5 μM) at the indicated time points or **(B)** 5-AzadC at the indicated concentrations for 48 h and the expression of HEXIM1 normalized to GAPDH was assessed using western blots. **(C)** C4-2 cells were treated with 5-AzadC (5 μM) at the indicated time points and the expression of DNMT1 normalized to GAPDH was assessed using western blots. **(D)** C4-2 cells were treated with Cladribine or Fludarabine at the indicated concentrations for 48 h and the expression of HEXIM1 normalized to GAPDH was assessed using western blots. **(E)** C4-2 cells were treated with 5-AzadC at the indicated concentrations for 48 h and the expression of pCHK1/CHK1 and pCHK2/CHK2 were assessed using western blots. Represented are blots cut into strips prior to blotting to minimize the amounts of antibodies required. In (A), (B), (C), and (E) figures are representative of at least 3 independent experiments. (D) is representative of 2 independent experiments. *$P<0.05$, **$P<0.01$ vs. Control.

Supplemental Figure 2. Treatment with ATM and ATR inhibitors attenuated 5-AzadC-induced pCHK1 and pCHK2 expression. (A) C4-2 cells were infected with control or ATR or ATM shRNA lentiviruses followed by puromycin selection. Cells were then treated with 5-AzadC (5 μM) for 48 h. Cell lysates were prepared and expression of ATR and ATM normalized to GAPDH were analyzed by western blot. Figures are representative of at least 3 independent experiments. *$P<0.05$ versus Control. **(B)** C4-2 and LNCaP cells were pre-treated with an ATR inhibitor, VE-822 (1 μM or 2 μM), or an ATM inhibitor, caffeine (1 μM or 2 μM) for 2 h, followed by 5-AzadC treatment (5 μM) for 48 h. Phospho-CHK1, total CHK1, phospho-CHK2, and total CHK2 levels were detected by western blot. Figures are representative of at least 3 independent experiments. *$P<0.05$ and **$P<0.01$ vs. Control. Represented are blots cut into strips prior to blotting to minimize the amounts of antibodies required.

Supplemental Figure 3. **CDK9 and NF-κB protein levels in cells treated with 5- AzadC, VE-822, and/or caffeine.** C4-2 and LNCaP cells were treated with VE-822 or caffeine for 2 h, followed by 5-AzadC for 2 h. Cells were **(A)** collected to assess CDK9 protein levels normalized by GAPDH using western blots, **(B)** processed for ChIP analyses of the occupancy of NF-κB on the coding region of HEXIM1, and **(C)** collected to assess NF-κB protein levels normalized by GAPDH using western blots. In (A) and (C), represented are blots cut into strips prior to blotting to minimize the amounts of antibodies required. In (B) input DNA was used as normalization control. IgG control was used as a negative control. Recruitment of CDK9 to the *HEXIM1* coding region was used as a positive control. Figures are representative of at least 3 independent experiments.

Supplemental Figure 4. **CDK9 and NF-κB protein levels in cells transduced with NFκB shRNA lentiviruses.** C4-2 and LNCaP cells were infected with control or NF-κB shRNA lentiviruses and selected with puromycin. Some cells were treated with 5-AzadC (5 μM, 2h) and then processed for western blotting to assess **(A)** NF-κB and **(B)** CDK9 protein levels normalized by GAPDH. Represented are blots cut into strips prior to blotting to minimize the amounts of antibodies required. Figures are representative of at least 3 independent experiments. *$P<0.05$ versus Control.

Supplemental Figure 5. 5-AzadC did not induce necroptosis. C4-2 cells were treated with 5-AzadC (5 μM) at the indicated time points and the expression of pMLKL normalized to total MLKL expression was assessed using western blots. Represented are blots cut into strips prior to blotting to minimize the amounts of antibodies required. Figures are representative of at least 3 independent experiments.

Supplemental Figure 6. **5-AzadC-induced HEXIM1 expression in breast cancer cells is mediated by ATM and NF-κB. (A)** MDA-MB-453 cells were infected with control or NF-κB shRNA lentiviruses followed by puromycin selection. Cell were then treated with 5-AzadC (5 μM) for 48 h. Cell lysates were prepared and expression of indicated proteins were analyzed by western blot. **(B)** MCF7 and T47D cells was treated with 5-AzadC at the indicated time points and the expression of HEXIM1 normalized to GAPDH was assessed using western blots. **(C)** MDA-MB-231 and MDA-MB-468 cells were treated with 5-AzadC (5 μM) for 48 h in the absence or presence of pre-treatment with ATM inhibitor, caffeine (2 μM). Expression of phospho-CHK2 normalized to total CHK2 was assessed by western blots. Represented are blots cut into strips prior to blotting to minimize the amounts of antibodies required. *P<0.01 versus Control. Figures are representative of at least 3 independent experiments.

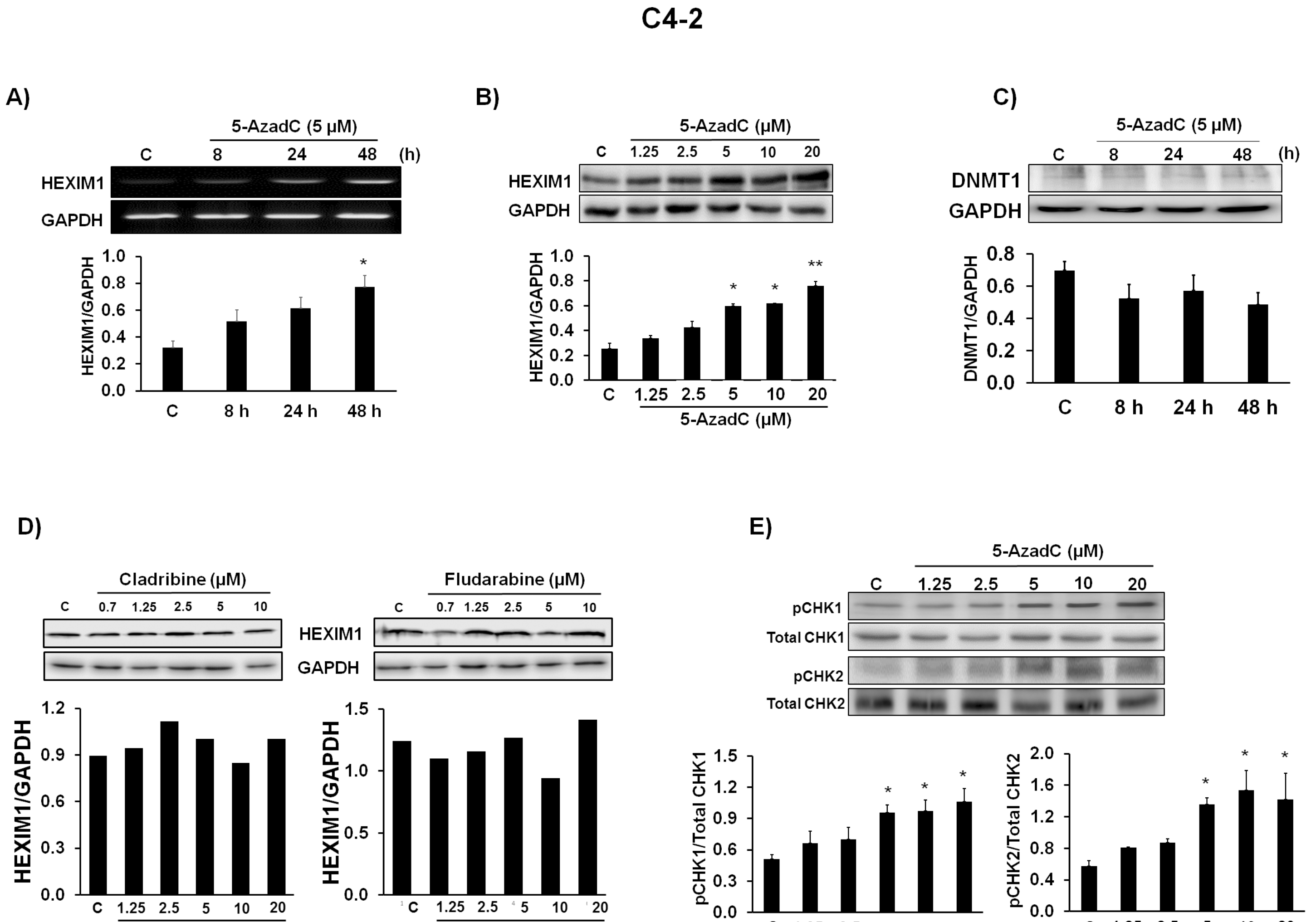

C4-2
A)
5-AzadC (5 μM)
C 8 24 48 (h)
HEXIM1
GAPDH
HEXIM1/GAPDH
C 8 h 24 h 48 h
B)
5-AzadC (μM)
C 1.25 2.5 5 10 20
HEXIM1
GAPDH
HEXIM1/GAPDH
C)
DNMT1
GAPDH
DNMT1/GAPDH
D)
Cladribine (μM)
C 0.7 1.25 2.5 5 10
Fludarabine (μM)
HEXIM1
GAPDH
HEXIM1/GAPDH
E)
pCHK1
Total CHK1
pCHK2
Total CHK2
pCHK1/Total CHK1
pCHK2/Total CHK2

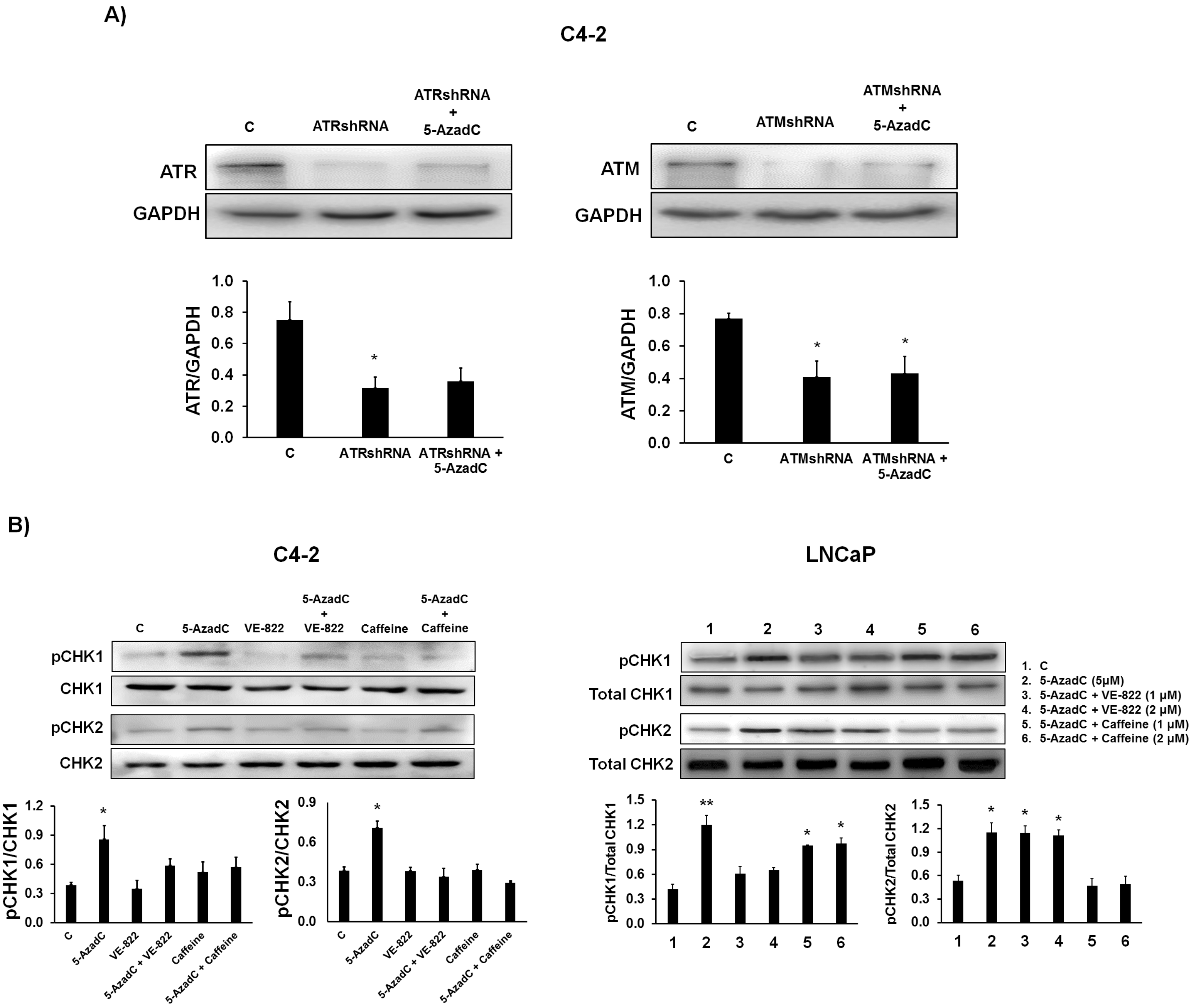

A)
C4-2
C
ATRshRNA
ATRshRNA + 5-AzadC
ATR
GAPDH
ATR/GAPDH
C
ATMshRNA
ATMshRNA + 5-AzadC
ATM
GAPDH
ATM/GAPDH
B)
C4-2
C
5-AzadC
VE-822
5-AzadC + VE-822
Caffeine
5-AzadC + Caffeine
pCHK1
CHK1
pCHK2
CHK2
pCHK1/CHK1
pCHK2/CHK2
LNCaP
1 2 3 4 5 6
pCHK1
Total CHK1
pCHK2
Total CHK2
1. C
2. 5-AzadC (5µM)
3. 5-AzadC + VE-822 (1 µM)
4. 5-AzadC + VE-822 (2 µM)
5. 5-AzadC + Caffeine (1 µM)
6. 5-AzadC + Caffeine (2 µM)
pCHK1/Total CHK1
pCHK2/Total CHK2

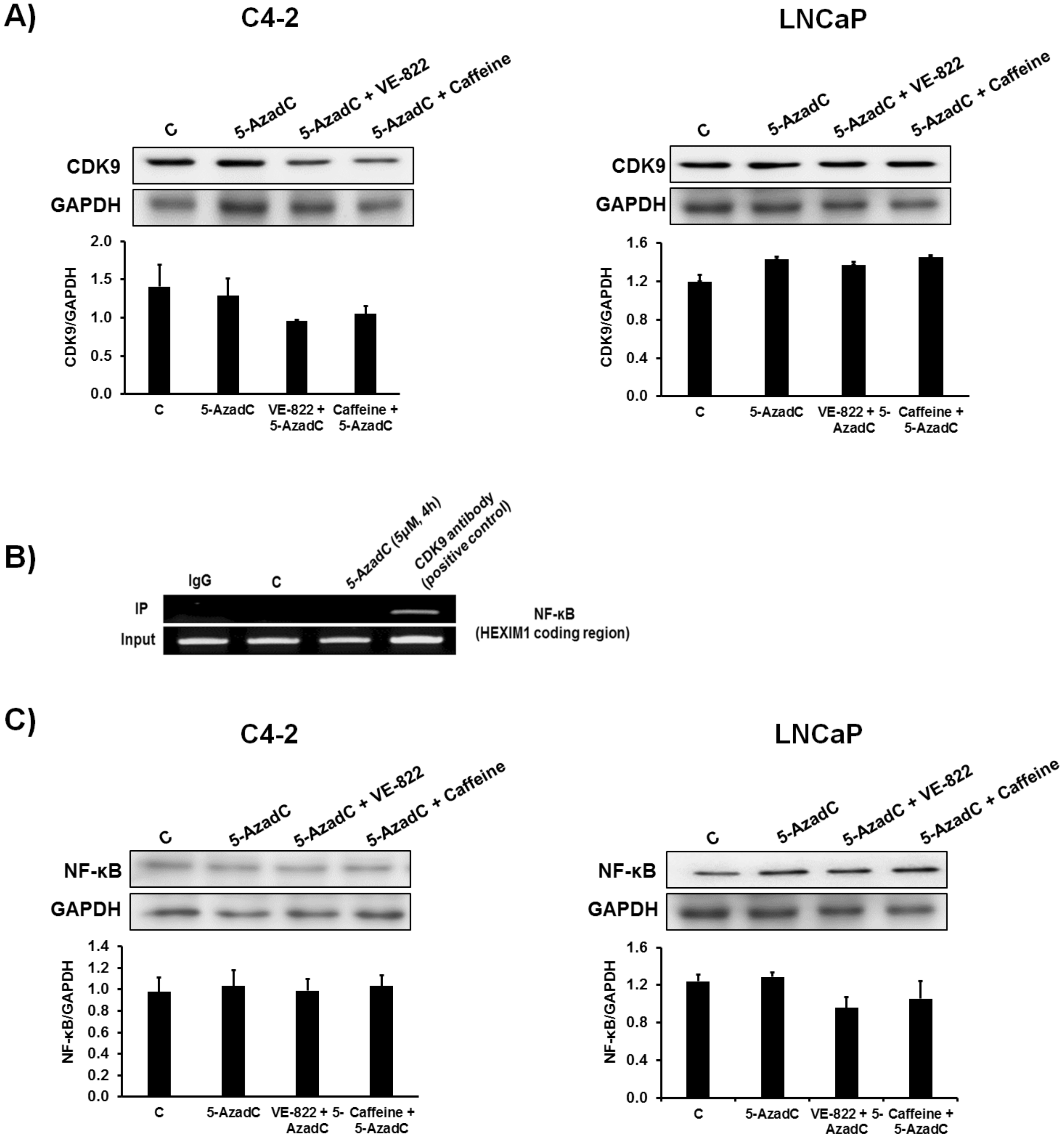

A)
C4-2
LNCaP
C
5-AzadC
5-AzadC + VE-822
5-AzadC + Caffeine
CDK9
GAPDH
CDK9/GAPDH
2.0
1.5
1.0
0.5
0.0
VE-822 + 5-AzadC
Caffeine + 5-AzadC
1.6
1.2
0.8
0.4
0.0
VE-822 + 5- AzadC
B)
IgG
C
5-AzadC (5μM, 4h)
CDK9 antibody (positive control)
IP
Input
NF-κB (HEXIM1 coding region)
C)
NF-κB
NF-κB/GAPDH
1.4
1.2
1.0
0.8
0.6
0.4
0.2
0.0

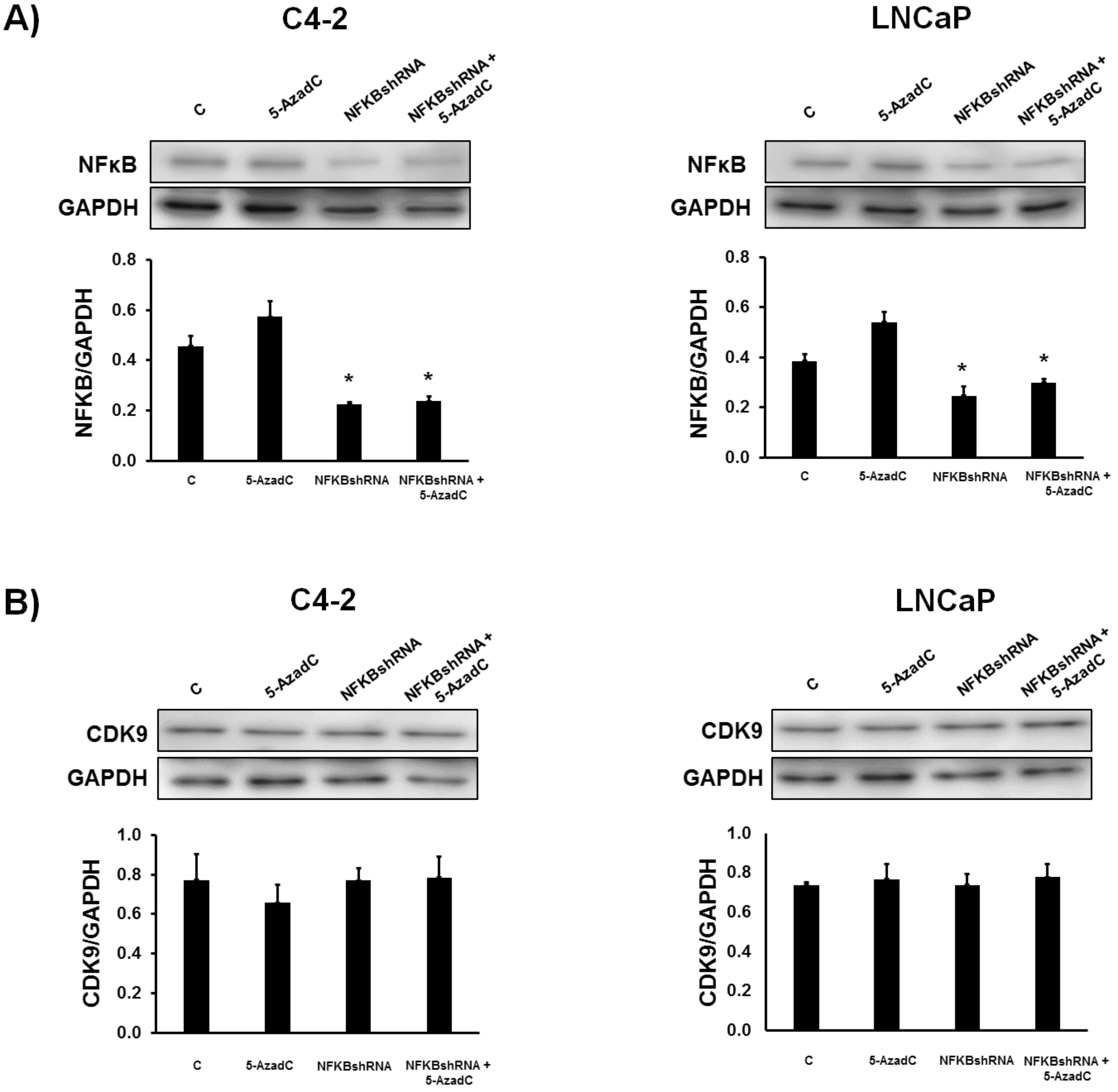
A)
C4-2
LNCaP
C
5-AzadC
NFKBshRNA
NFKBshRNA +
5-AzadC
NFκB
GAPDH
NFKB/GAPDH
0.0
0.2
0.4
0.6
0.8
*
B)
C4-2
LNCaP
CDK9
GAPDH
CDK9/GAPDH
1.0

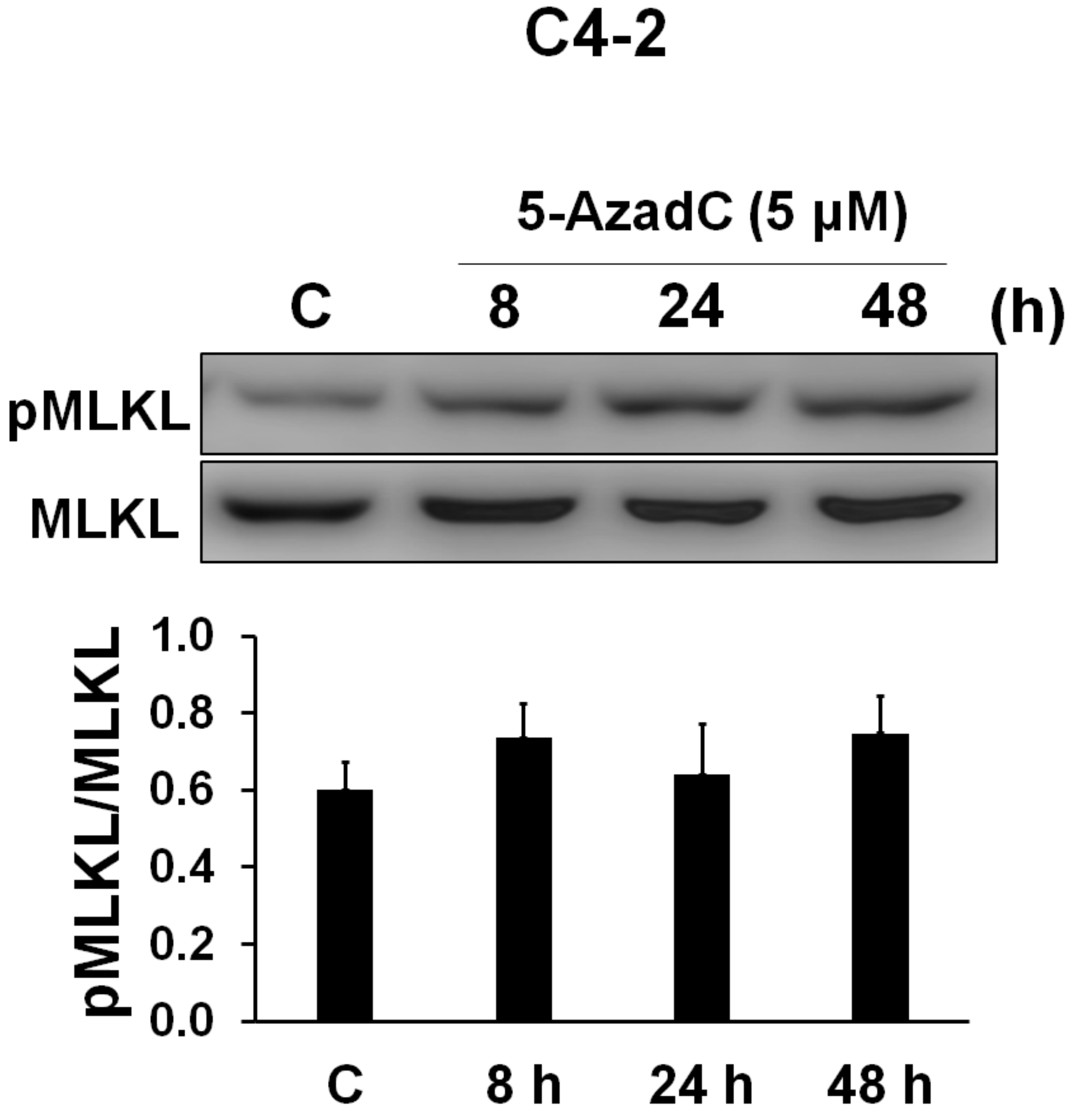

C4-2
5-AzadC (5 μM)
C
8
24
48
(h)
pMLKL
MLKL
pMLKL/MLKL
1.0
0.8
0.6
0.4
0.2
0.0
C
8 h
24 h
48 h

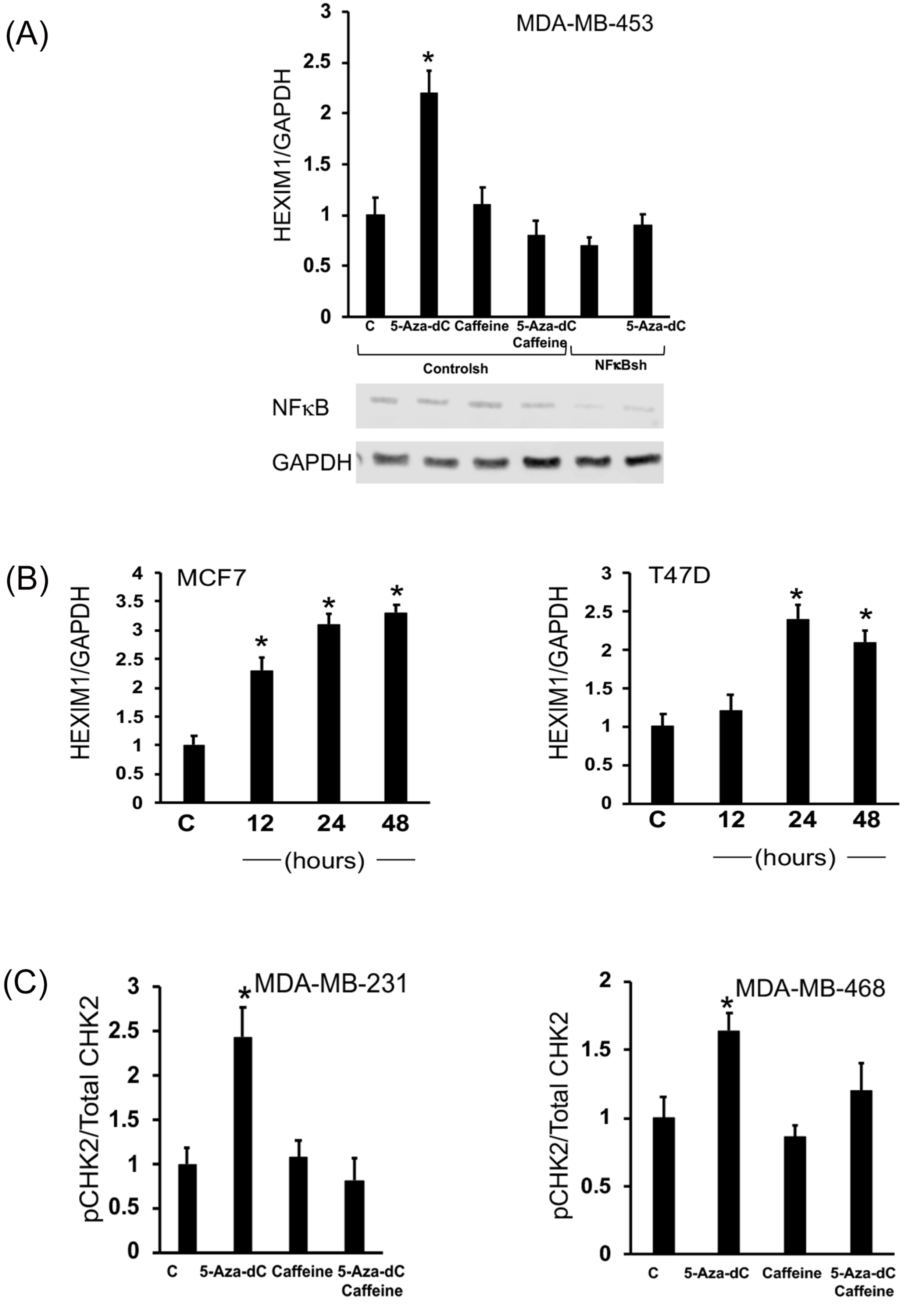
(A)
MDA-MB-453
HEXIM1/GAPDH
3
2.5
2
1.5
1
0.5
0
*
C
5-Aza-dC
Caffeine
5-Aza-dC Caffeine
5-Aza-dC
Controlsh
NFκBsh
NFκB
GAPDH
(B)
MCF7
HEXIM1/GAPDH
4
3.5
3
2.5
2
1.5
1
0.5
0
C
12
24
48
(hours)
T47D
HEXIM1/GAPDH
3
2.5
2
1.5
1
0.5
0
C
12
24
48
(hours)
(C)
MDA-MB-231
pCHK2/Total CHK2
3
2.5
2
1.5
1
0.5
0
C
5-Aza-dC
Caffeine
5-Aza-dC Caffeine
MDA-MB-468
pCHK2/Total CHK2
2
1.5
1
0.5
0
C
5-Aza-dC
Caffeine
5-Aza-dC Caffeine

Figure 1

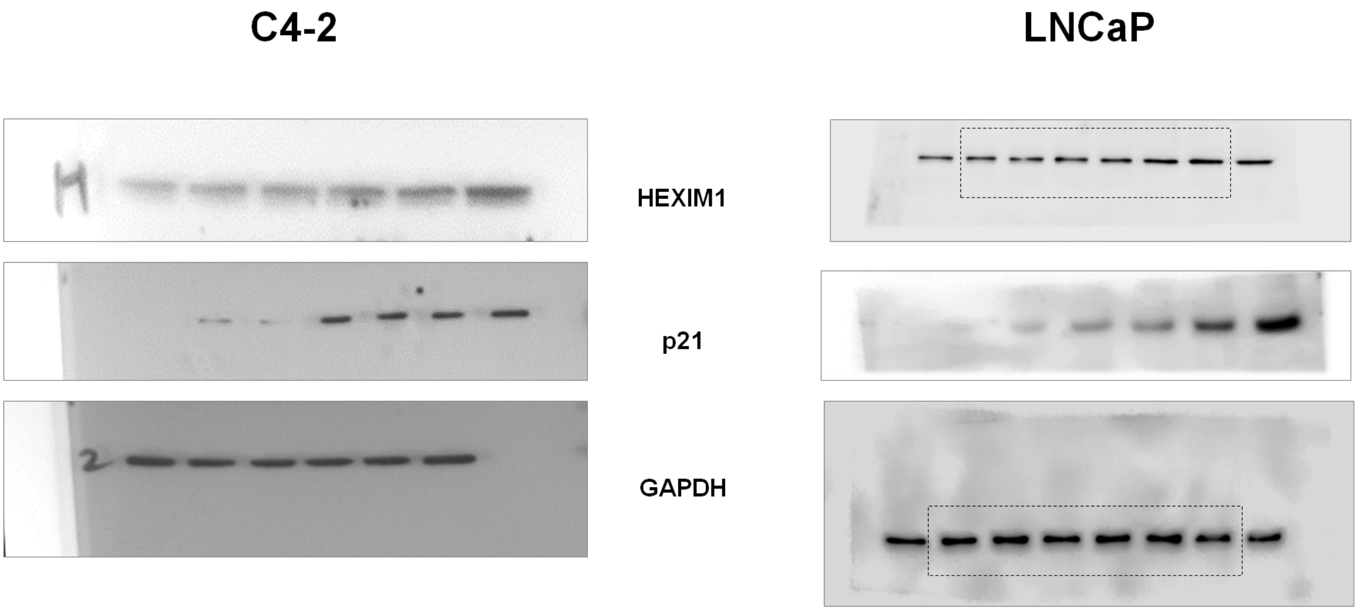

Figure 2

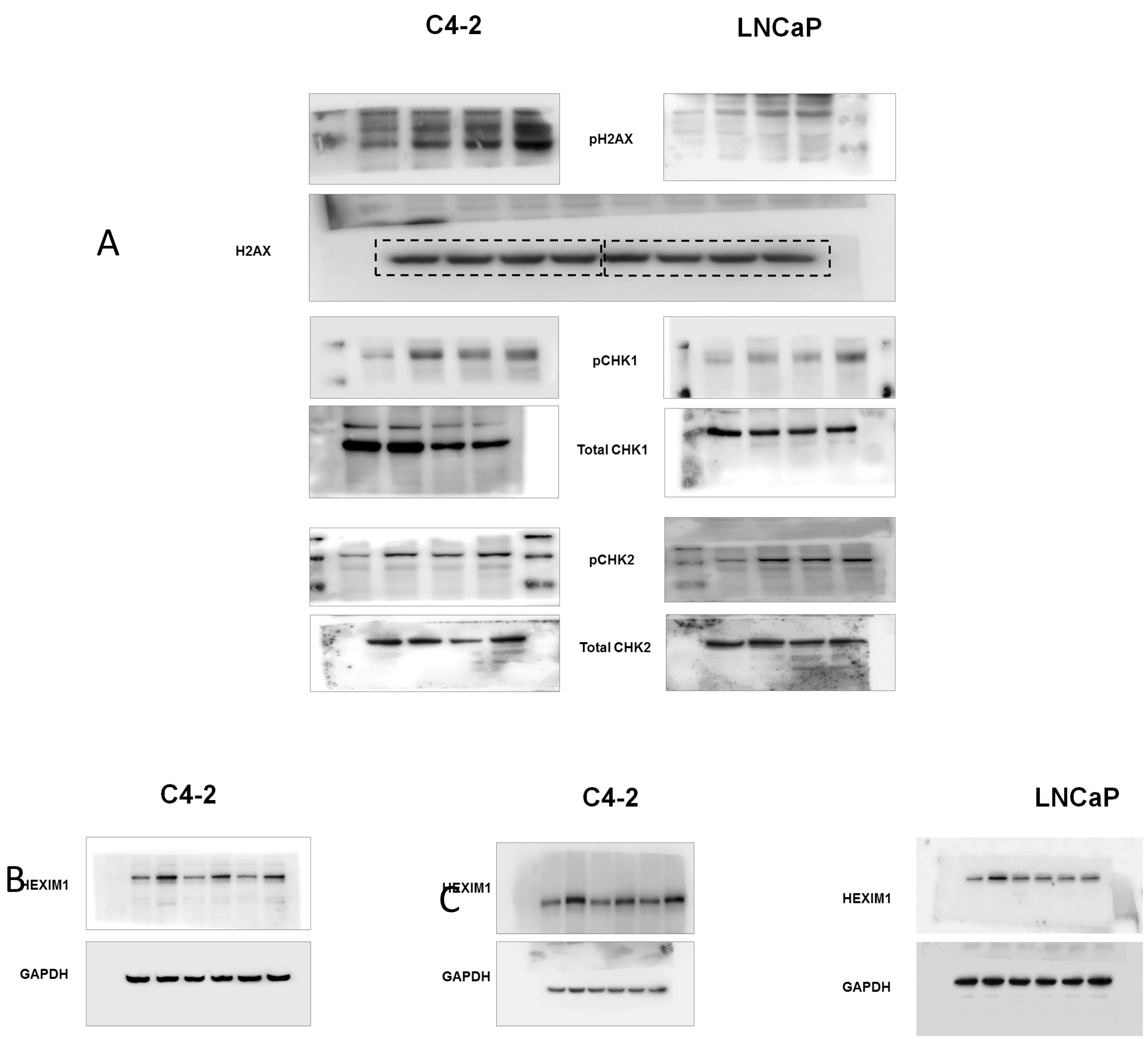
C4-2
LNCaP
A
pH2AX
H2AX
pCHK1
Total CHK1
pCHK2
Total CHK2
C4-2
C4-2
LNCaP
B
HEXIM1
GAPDH
C
HEXIM1
GAPDH
HEXIM1
GAPDH

Figure 3

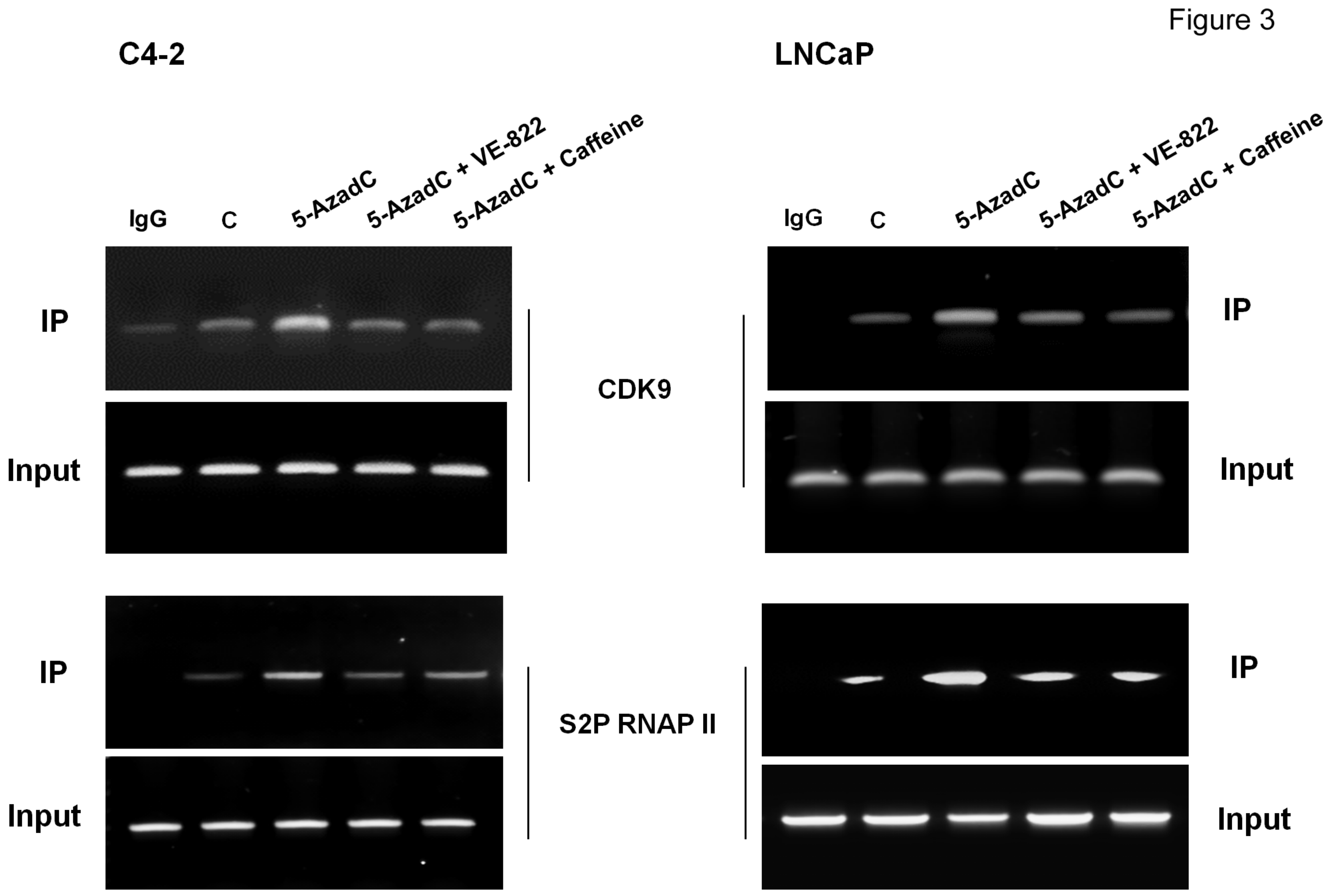
Figure 3
C4-2
LNCaP
IgG
C
5-AzadC
5-AzadC + VE-822
5-AzadC + Caffeine
IP
Input
CDK9
S2P RNAP II

Figure 4

A

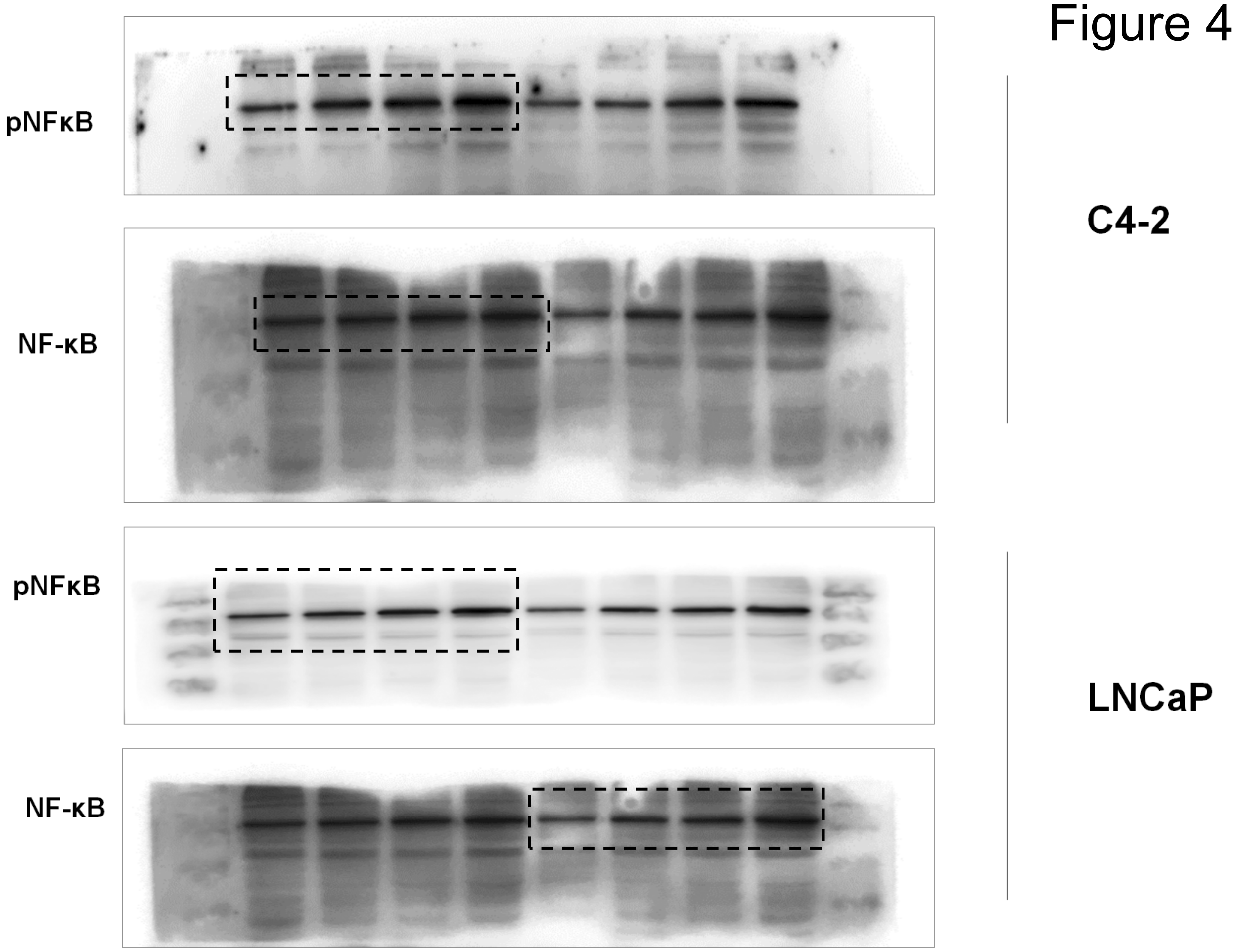

C

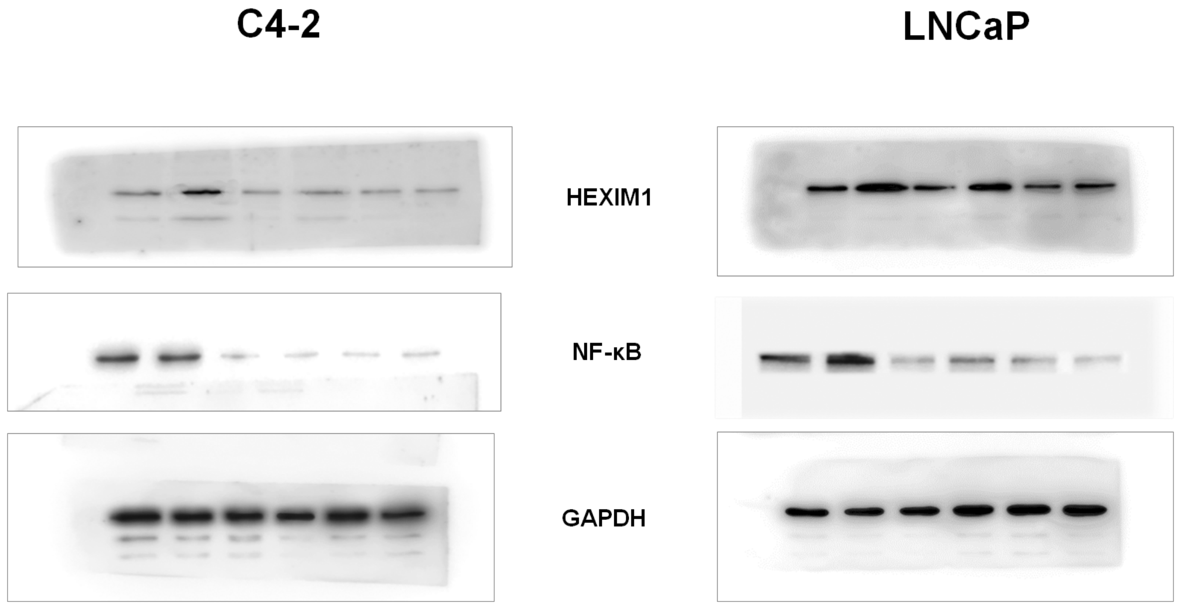

Figure 4

(B)

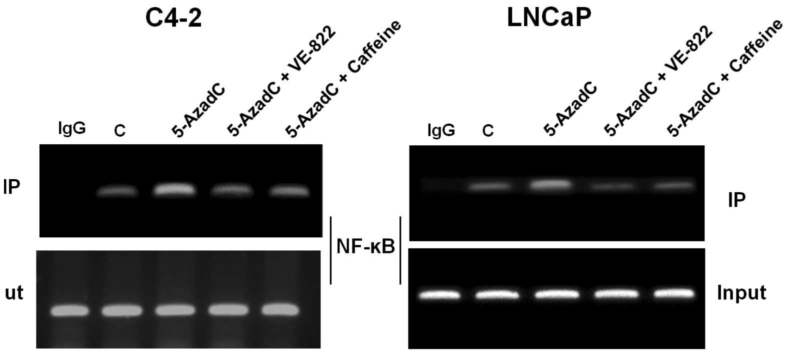
C4-2
LNCaP
IgG
C
5-AzadC
5-AzadC + VE-822
5-AzadC + Caffeine
IP
ut
NF-κB
IP
Input

(D)

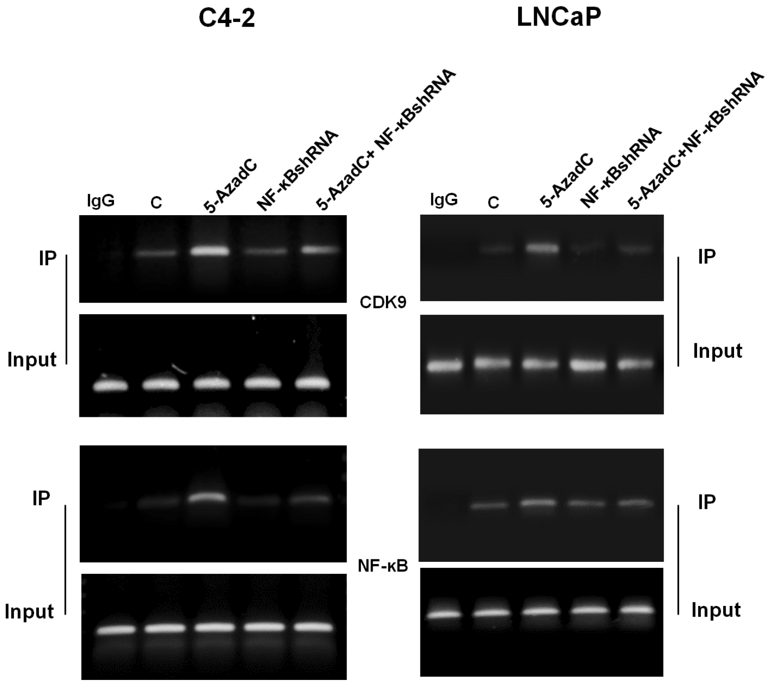
C4-2
LNCaP
IgG
C
5-AzadC
NF-κBshRNA
5-AzadC+ NF-κBshRNA
5-AzadC+NF-κBshRNA
IP
Input
CDK9
NF-κB

Figure 5A

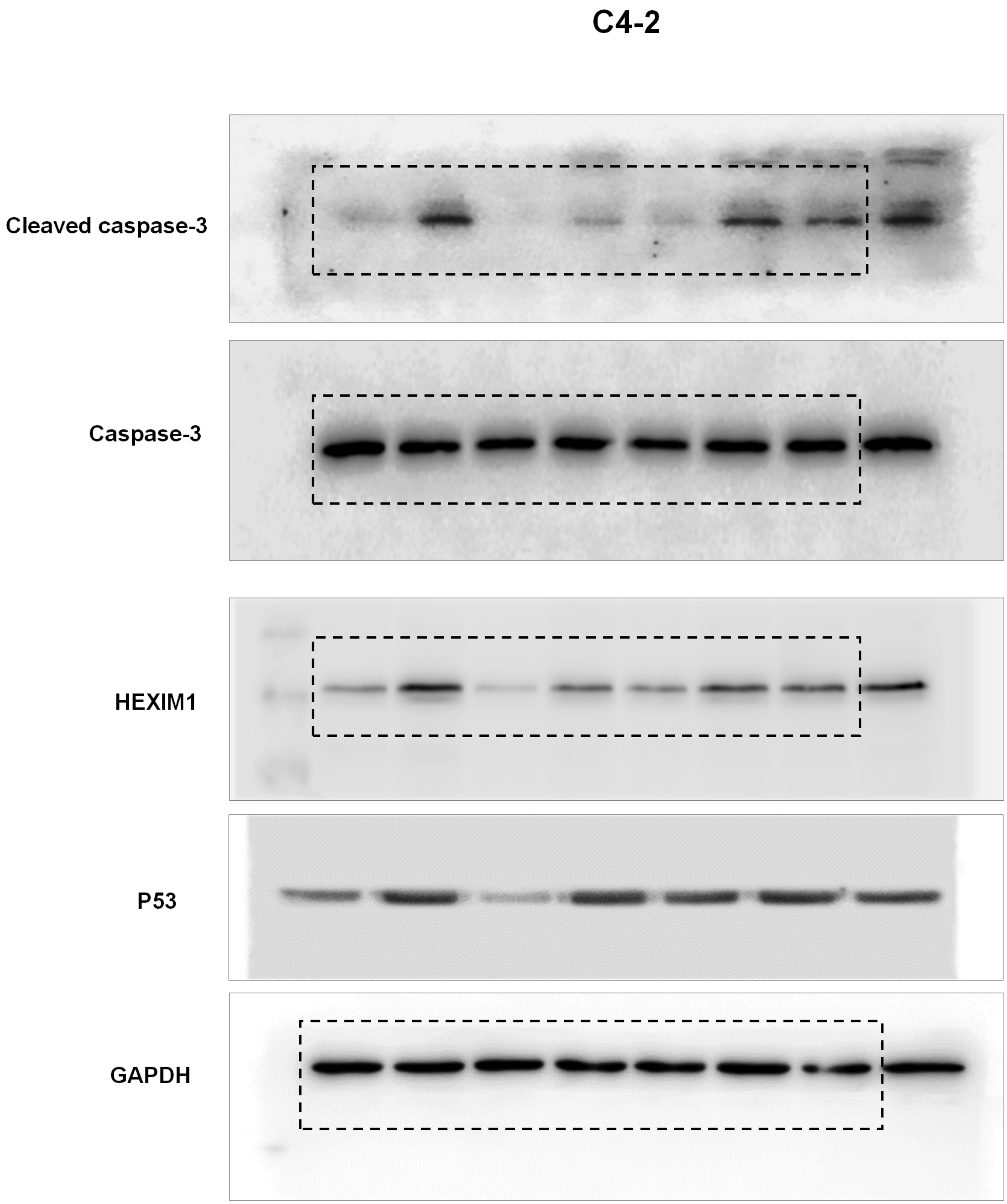

Figure 6A

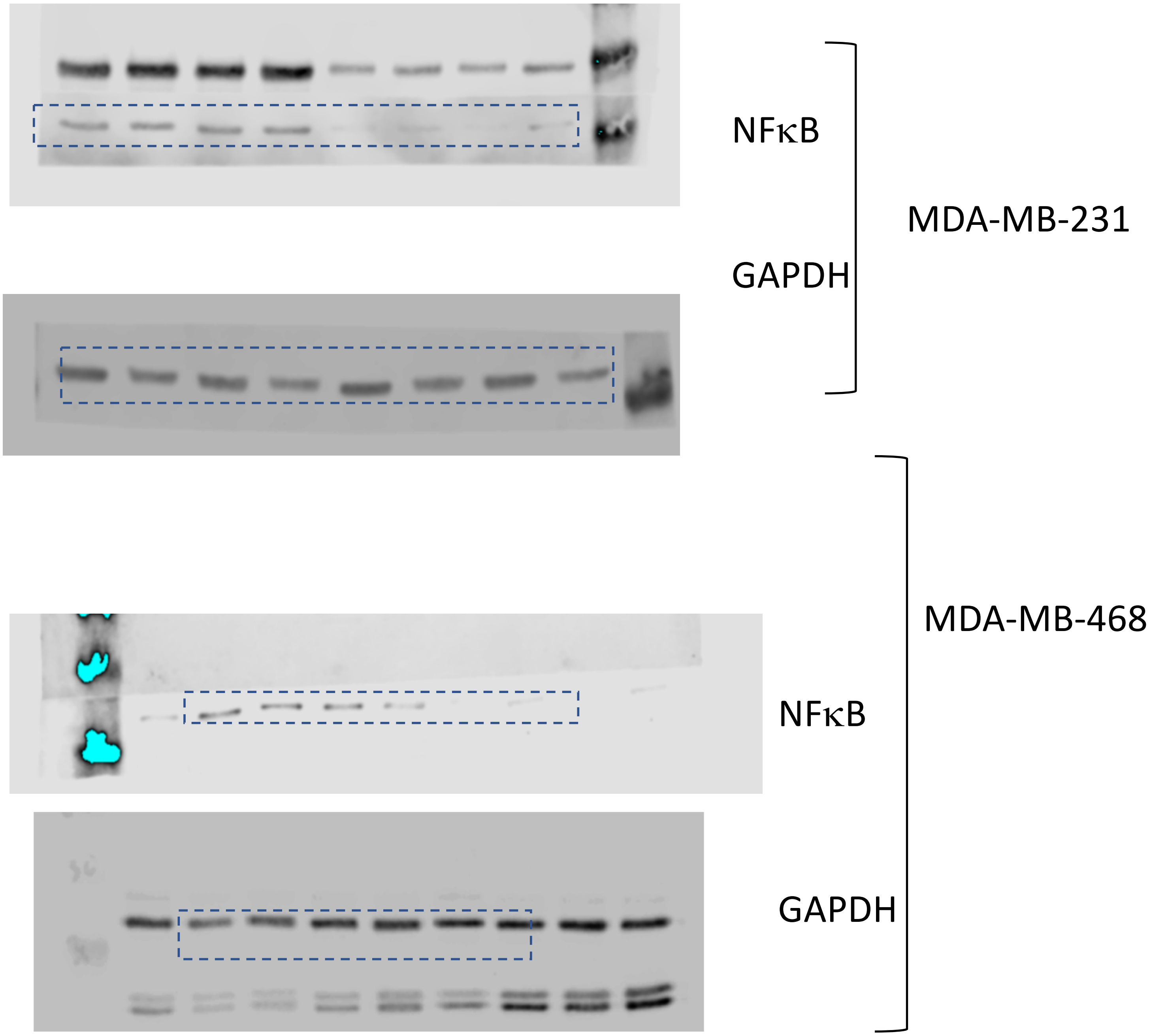

Figure 6B

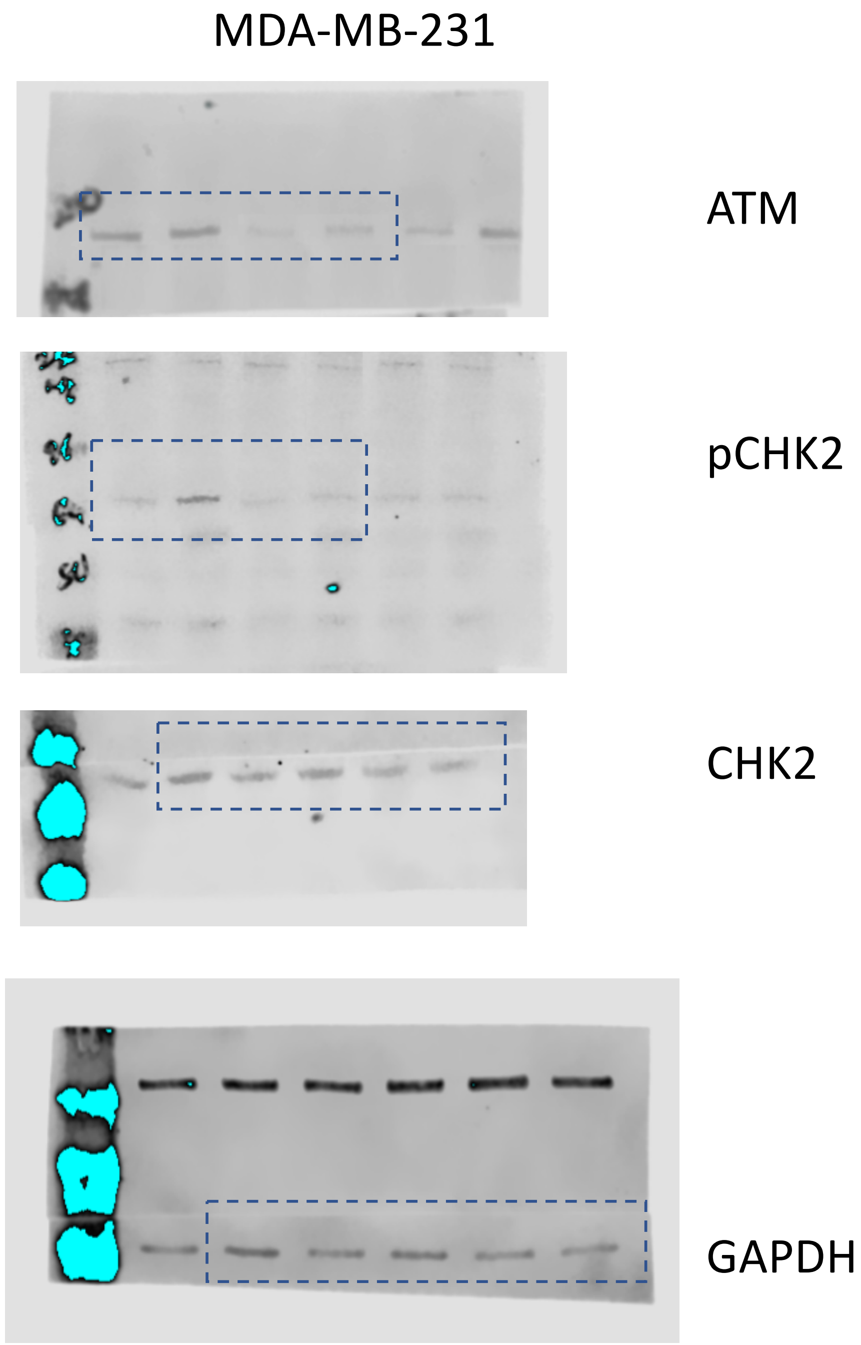

Figure 6B

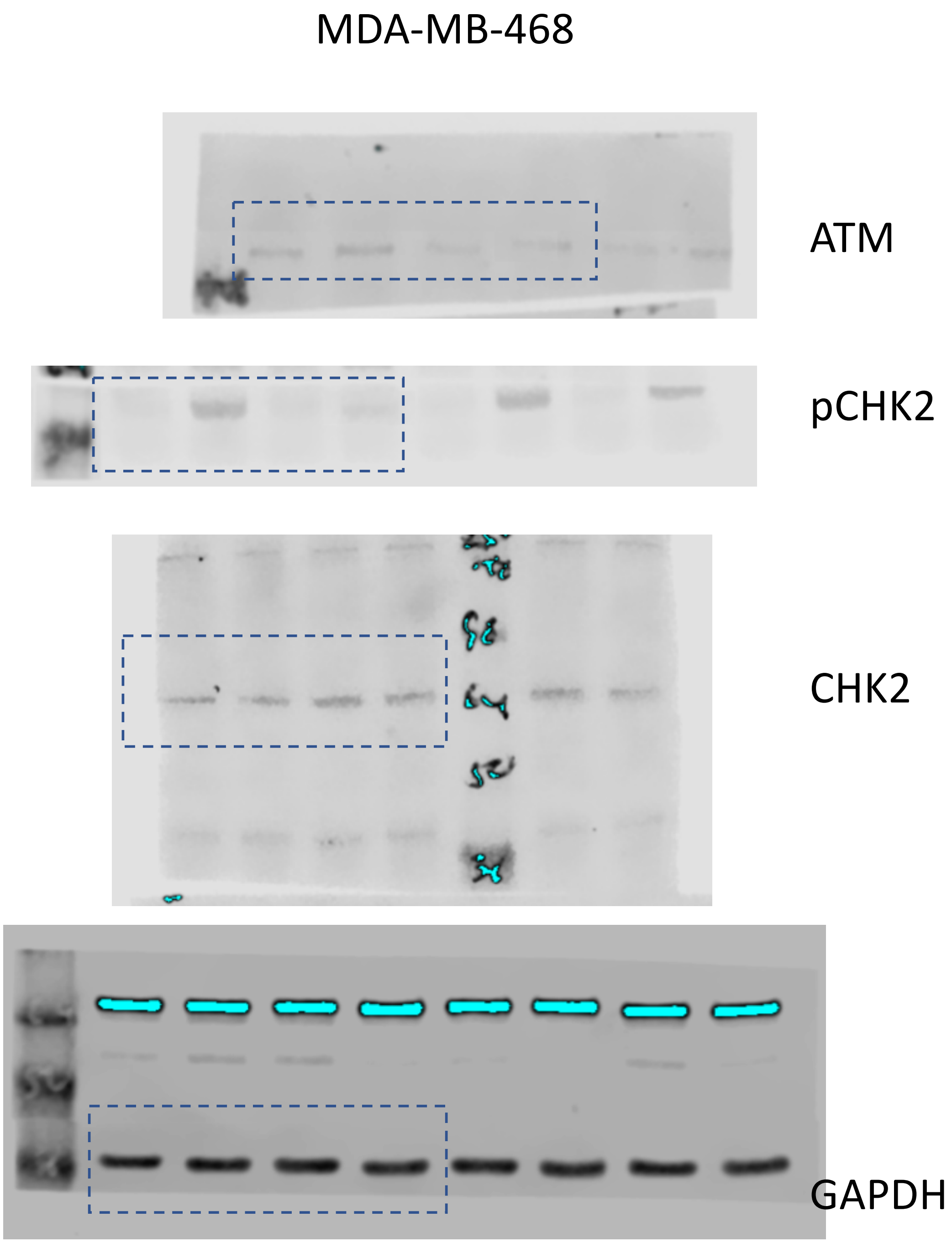

Figure 6C

MDA-MB-231

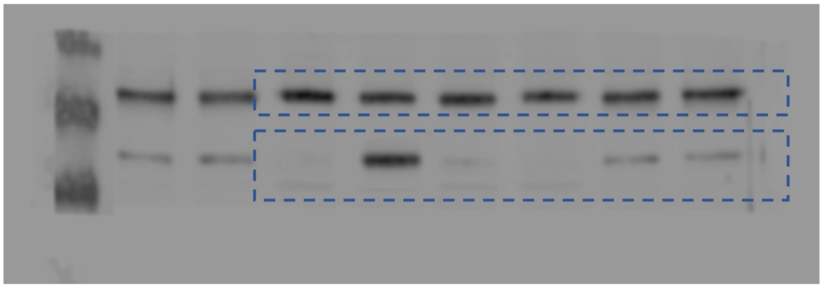

MDA-MB-468

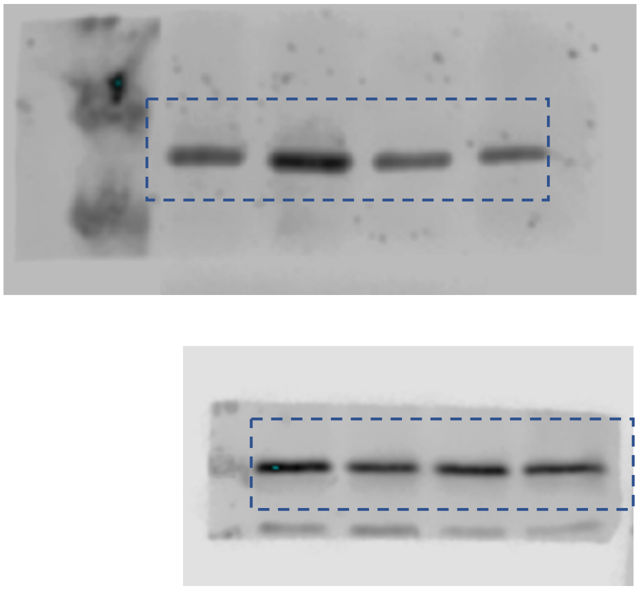

Supplementary Figure 1

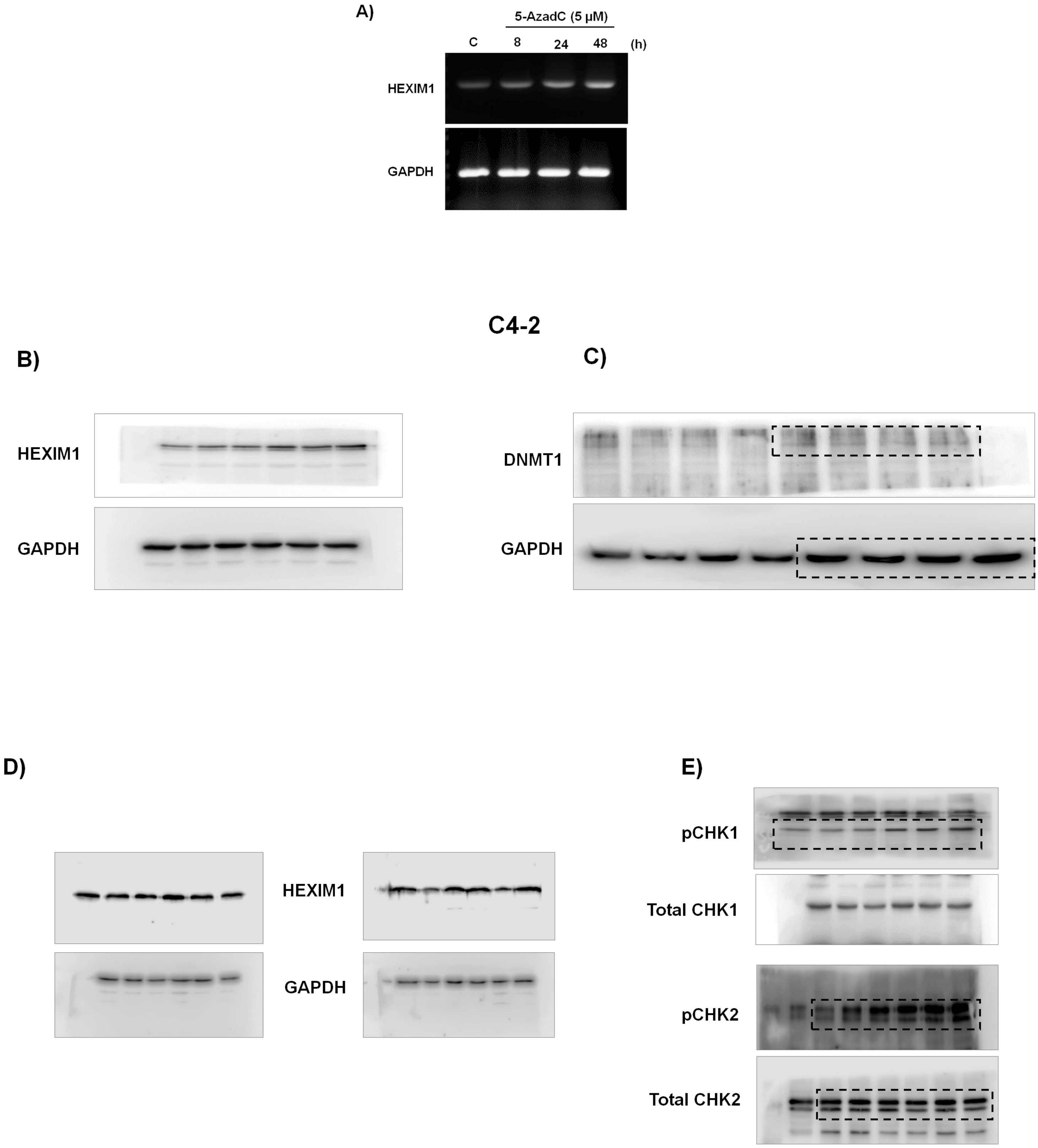

Supplementary Figure 2

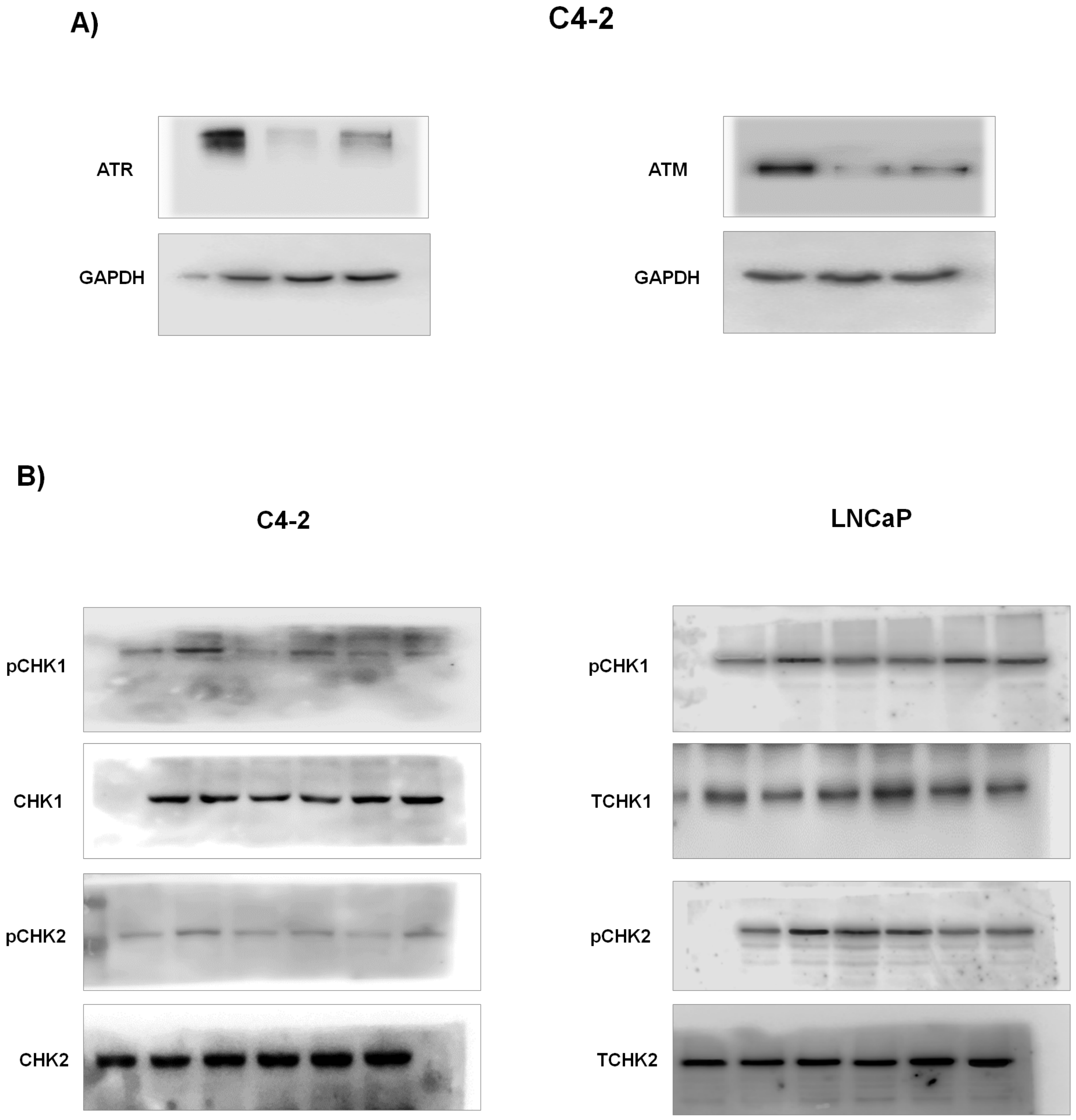

Supplementary Figure 3

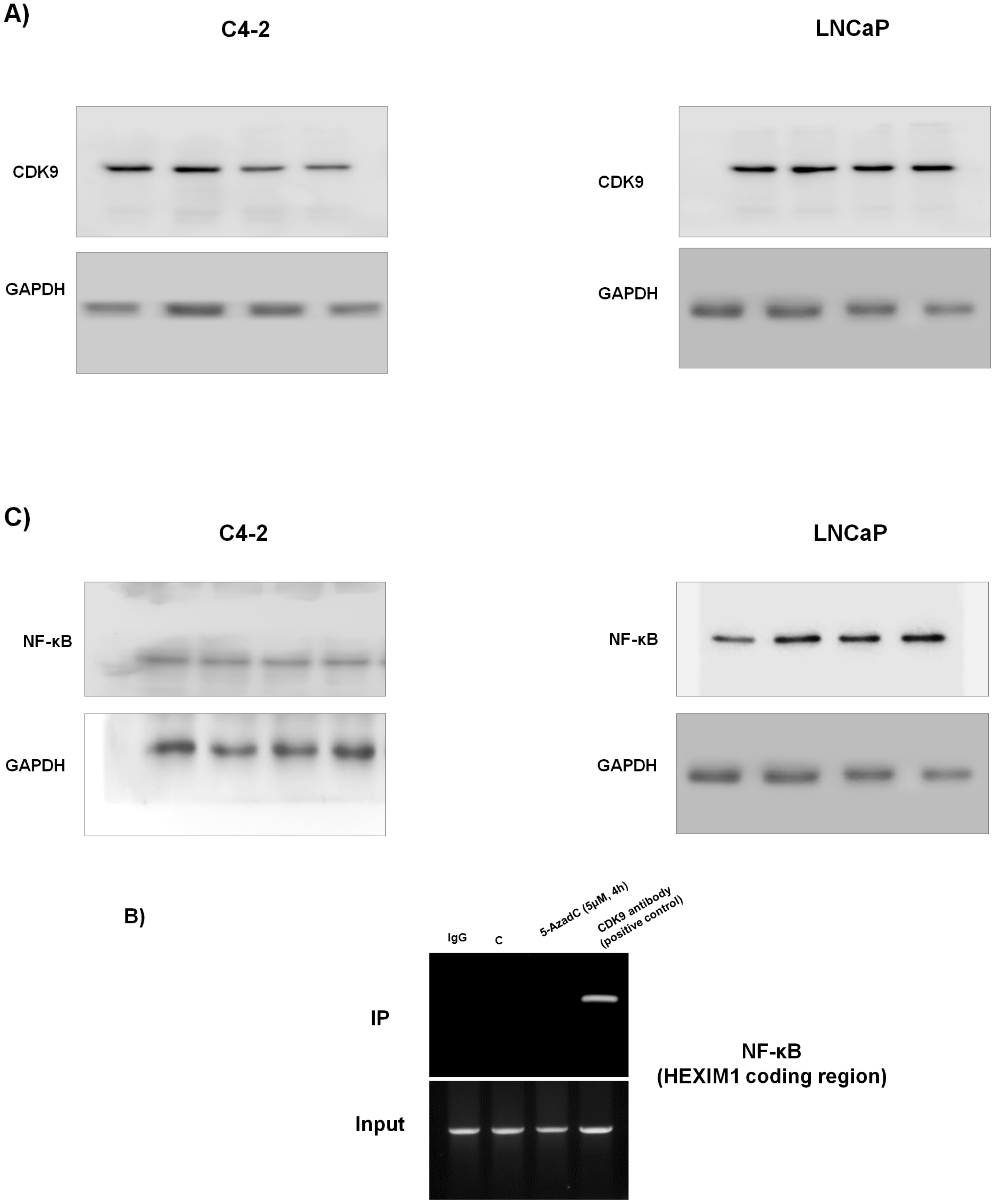

Supplementary Figure 4

A)

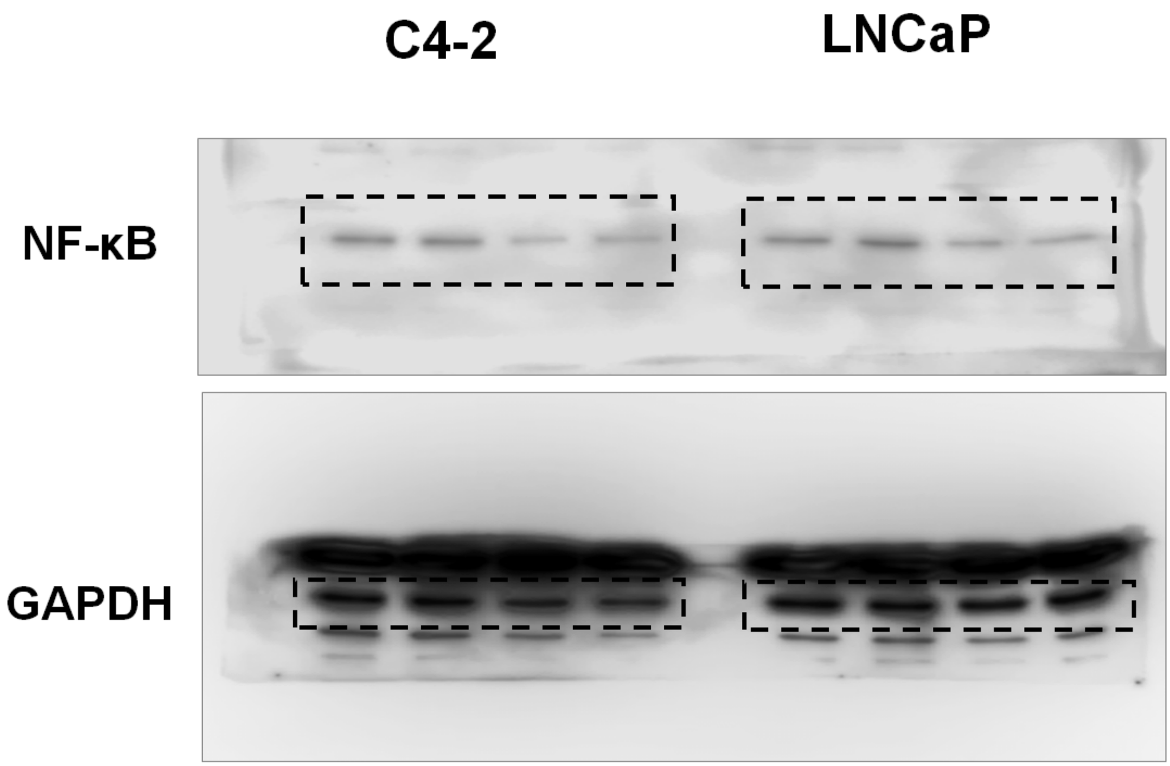

B)

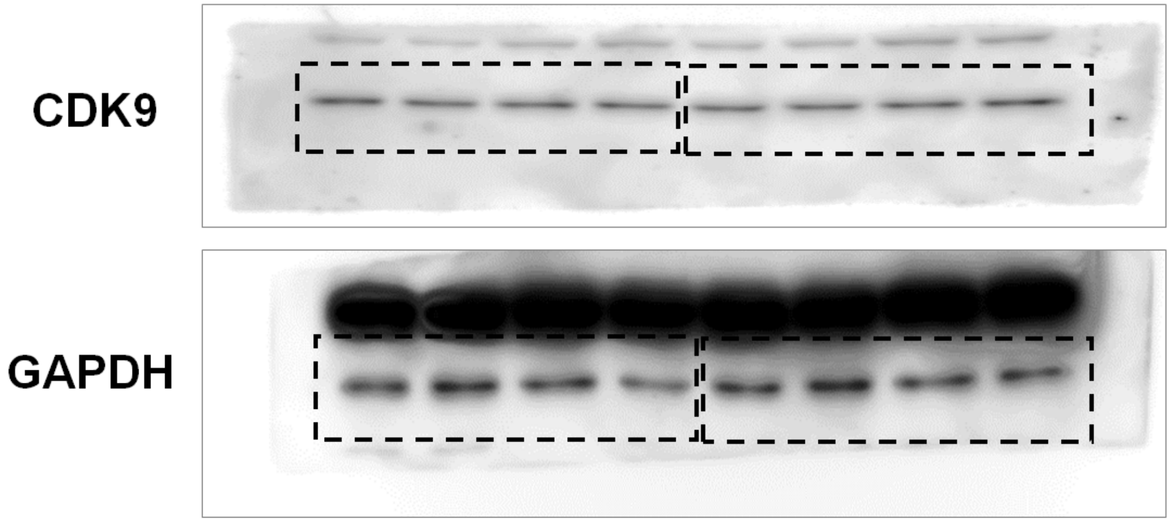

Supplementary Figure 5

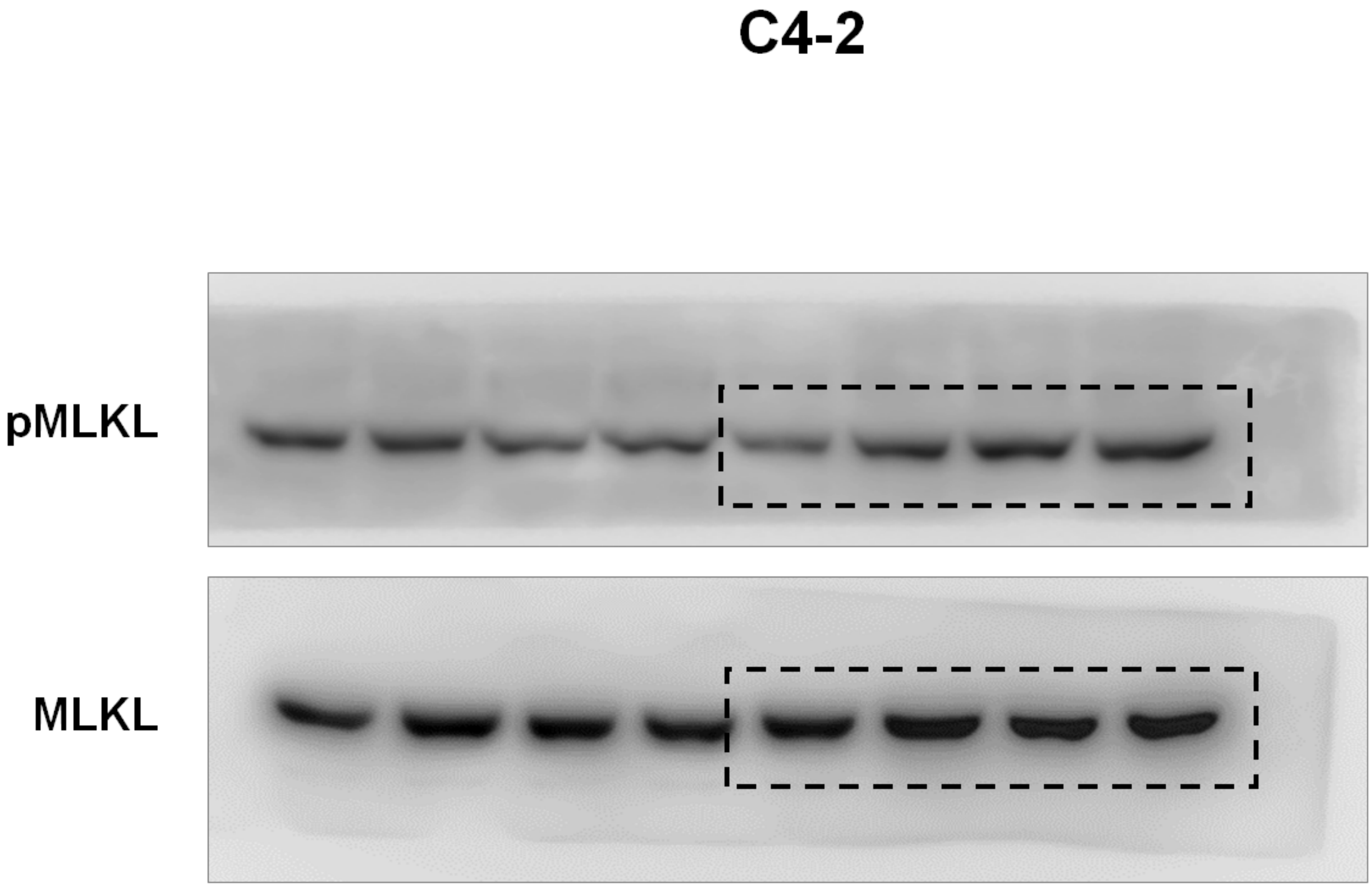

Supplementary Figure 6

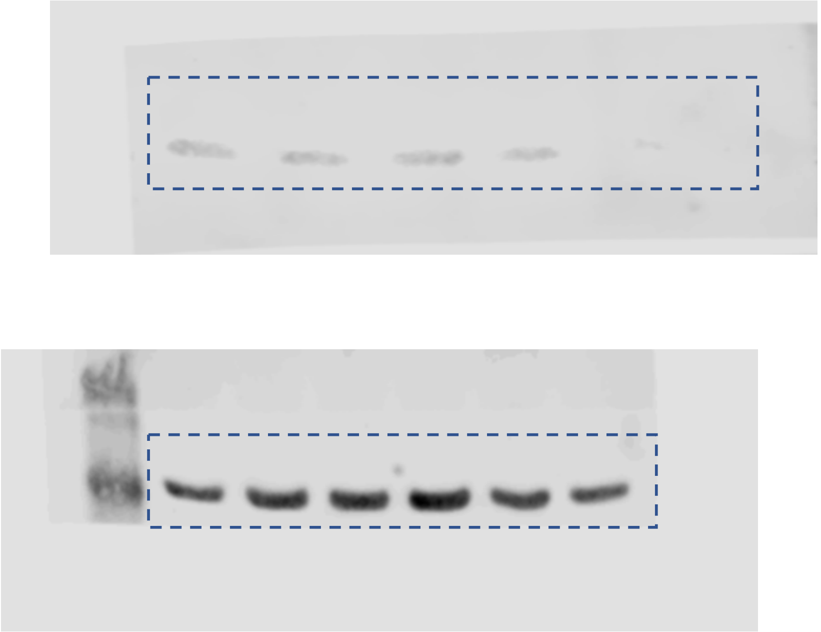

NFκB

GAPDH
